# A spatial genome aligner for resolving chromatin architectures from multiplexed DNA FISH

**DOI:** 10.1038/s41587-022-01568-9

**Published:** 2023-01-02

**Authors:** Bojing Blair Jia, Adam Jussila, Colin Kern, Quan Zhu, Bing Ren

**Affiliations:** 1 Bioinformatics and Systems Biology Graduate Program, University of California San Diego, La Jolla, CA USA; 2 Medical Scientist Training Program, University of California San Diego, La Jolla, CA USA; 3 Department of Cellular and Molecular Medicine, Center for Epigenomics, University of California San Diego, La Jolla, CA USA; 4 Ludwig Institute for Cancer Research, La Jolla, CA USA; 5 Institute of Genomic Medicine, Moores Cancer Center, School of Medicine, University of California San Diego, La Jolla, CA USA

**Keywords:** Gene regulation, Image processing

## Abstract

Multiplexed fluorescence in situ hybridization (FISH) is a widely used approach for analyzing three-dimensional genome organization, but it is challenging to derive chromosomal conformations from noisy fluorescence signals, and tracing chromatin is not straightforward. Here we report a spatial genome aligner that parses true chromatin signal from noise by aligning signals to a DNA polymer model. Using genomic distances separating imaged loci, our aligner estimates spatial distances expected to separate loci on a polymer in three-dimensional space. Our aligner then evaluates the physical probability observed signals belonging to these loci are connected, thereby tracing chromatin structures. We demonstrate that this spatial genome aligner can efficiently model chromosome architectures from DNA FISH data across multiple scales and be used to predict chromosome ploidies de novo in interphase cells. Reprocessing of previous whole-genome chromosome tracing data with this method indicates the spatial aggregation of sister chromatids in S/G2 phase cells in asynchronous mouse embryonic stem cells and provides evidence for extranumerary chromosomes that remain tightly paired in postmitotic neurons of the adult mouse cortex.

## Abstract (web summary)

The architectures of chromatin fibers are resolved using a spatial genome aligner.

## Main

Eukaryotic chromosomes undergo dramatic compaction and decompaction in the life cycle of a cell, and the dynamic chromosomal structure plays an integral role in a range of nuclear processes such as DNA replication, recombination, repair and gene transcription^1–5^. In interphase nuclei, different chromosomes generally occupy separate territories with limited intermingling^6^. Within each chromosomal territory, the chromatin fibers are organized into compartments and domains^7–11^ driven in part by the ATP-dependent motor protein complex and loop extruder cohesin^12–16^. The complex chromatin structures enable juxtaposition of remote DNA in space and subsequent transcriptional activation of genes by distal enhancers^5,17–19^. Disruption of chromatin structures underlies a score of pathologies ranging from limb malformations, oncogenesis, to heart disease^20–23^. Delineating how chromatin fibers are folded in the nucleus is therefore of fundamental importance for the study of gene regulation and other nuclear processes in health and disease.

Multiplexed DNA fluorescence in situ hybridization (M-DNA-FISH) is a widely used imaging technique for the study of chromatin structure in eukaryotic cells^24–43^. These technologies can identify tens to thousands of genomic loci in the nucleus, permitting potential folding patterns of chromosomes to be detected. They achieve this through the use of barcodes that, when decoded via serial hybridization of fluorescent probes, enable sequence-specific identification and, thus distinction, of genomic loci; in diffraction-limited imaging the chromatin fiber connecting them is not visualized and must be inferred. The inference of physical connection between two discrete signals is the most salient problem facing chromatin imaging so far. Early efforts to multiplex DNA FISH often found one, three or four fluorescent signals emanating from one genomic region in a diploid cell line expected to produce two signals^35,36^. Biological copy number variation, chromosomal intermingling as well as poor probe hybridization have been acknowledged to explain missing signals^31,32,35^, sister chromatids and aneuploidy as well as imaging noise have been acknowledged to explain extra signals^31,34,35^. If both noise and biological variation can explain any observed scenario, chromatin fibers cannot be naïvely traced by connecting the first immediate spot. In fact, this uncertainty around imaging has led some to forgo tracing altogether and instead tabulate proximal pairs of imaged loci for bulk analysis^31^. When noise appears indistinguishable from true imaged genomic loci, and biological variation at the single-cell level confounds expectation, reconstruction of chromatin fibers remains an intractable computational problem.

In the current benchmark for chromatin tracing, the tracing problem is simplified with assumptions about copy number and with emphasis on the optical quality of detected signals. An expectation–maximization algorithm (E–M) is first tasked to find *k* chromatin fibers corresponding to a *k*-ploid cell^28,30^. Repeated *k*-times per cell, candidate fluorescence spots corresponding to a genomic region are scored based on signal intensity and proximity to a moving average of downstream selected spots, as well as upstream selected spots and proximity to a chromosome center (that is, the aggregate of many fluorescent loci) determined by *k*-means clustering^28,30^. Implicit in this approach are two strong assumptions: that the brightness is a measure of detection confidence and that the copy number of a DNA segment is fixed and known beforehand. However, background fluorescence, nonspecific probe binding and even hot pixels can frequently emit similarly intense focal signals indistinguishable from the true signal. Additionally, looking for a fixed number of chromosomes may fail to capture true biological copy number variations and aneuploidy. To disambiguate these scenarios, we need a new framework for chromatin tracing that leverages yet unused information, improving on the above heuristics.

Here we present a spatial genome aligner that considers the above challenges. We reason that while the shape of chromatin fiber is highly variable, it is subject to spatial constraints dictated by polymer physics^44,45^. In addition to considering optical quality of signals, our algorithm aims to select true fluorescence spots corresponding to a DNA locus from a number of candidates by picking the one that best conforms to a reference polymer model of chromatin. Briefly, these restrictions are the genomic distances between two discrete sequence-specific labeled loci, which should be proportional to the square of its spatial separation. We use the Gaussian chain polymer model to estimate an expected spatial distance given a genomic distance, and compare the observed spatial distance in imaging to this estimated spatial distance as a test of physical likelihood^44^. We attempted to evaluate the accuracy of the spatial genome aligner by comparing pairwise distances discovered by tracing against pairwise contact frequencies discovered by Hi-C. We find that our spatial genome aligner can recapitulate patterns of chromatin organization found in Hi-C at multiple genomic length scales (5 kilobase (kb), 25 kb and 1 megabase (Mb)). We note circumstances where chromatin folding may not completely adhere to this Gaussian chain model, and chromatin structure may be influenced by factors including but not limited to chromatin remodelers, gene expression, proximity to nuclear structures and certain chromatin loops that in part or in sum evade prediction by the Gaussian chain, despite our model’s best effort to permit flexibility. The genomic footprint of the oligos tiling at a target site, as well as transcriptional state of the target site, may also influence measurement. We also acknowledge that diffraction-limited imaging currently lacks the sensitivity to resolve individual DNA oligos and needs to ‘merge’ the cumulative signals of multiple oligos. Image smoothing may inadvertently fuse signals belonging to separate chromatin fibers; conversely, sample prep and large genomic footprint may potentially fragment one signal. We further note that extrapolation of persistence length of DNA to chromatin scale remain challenging. Therefore, we removed one free variable by fixing the persistence length (*l*_p_ = 150 bp) and fitting a variable genomic-to-spatial distance scale (*τ*). We find our spatial genome aligner uncovers more chromatin fibers than previously reported in published datasets, and infer that these extra fibers may in fact be sister chromatids. We show that each pair of putative sister chromatids usually resides in a spatially separate chromosome territory, but in roughly 2% of replicating cells both pairs of sister chromatids coalesce to interact in one convergent territory. We go on to apply our spatial genome alignment to previous chromatin tracing data generated from mouse cortical excitatory neurons, where we uncover patterns of spatial organization of extranumerary chromosomes inside the nucleus.

## Results

### Spatial genome alignment

Chromosomes are linear, flexible polymers that take on convoluted structures inside the nucleus. One simple but robust model for the spatial configuration of flexible polymers is a Gaussian chain^44^. In this model, the polymer is represented as a chain of successive monomers, linked by bonds of approximately constant length *b*. Each successive monomer is allowed to freely rotate with respect to each other. Transitioning from one monomer to another along the polymer chain is to take one step in a three-dimensional (3D) random walk. For any two monomers *i* and *j* on this chain, the probability they are separated by a vector distance *R*_*ij*_ follows a Gaussian distribution (hence, Gaussian chain)^44^:

1 $$P\left( {R_{ij},n} \right) = \frac{1}{{\left( {\frac{{2\pi }}{3}nb^2} \right)^{3/2}}}{\mathrm{e}}^{ - \left( {\frac{{3R_{ij}^2}}{{2nb^2}}} \right)}$$

where *n* is the number of bonds, each of length *b*, separating two monomers *i* and *j*.

For a chain with *N* bonds, the likelihood of the entire chain (also known as the conformational distribution function, CDF) is the product of all bond probabilities on the chain^44^:

2 $$\Psi \left( {\{ R_N\} } \right) = \mathop {\prod }\limits_{n = 1}^N \frac{1}{{\left( {\frac{{2\pi }}{3}b^2} \right)^{3/2}}}{\mathrm{e}}^{ - \left( {\frac{{3R_n^2}}{{2b^2}}} \right)}$$

In multiplexed DNA FISH experiments, we recognize entire chromosomes are labeled at discrete positions, analogous to discrete monomers on a Gaussian chain. Furthermore, these discrete loci are interspaced at regular genomic intervals (for example, 1 Mb), akin to the constant bond length *b* that separate monomers on the model chain. We hypothesized that at large genomic length scales, we can model DNA conformation with a Gaussian chain in which the bond length *b* can be estimated from the genomic distance separating two loci^44,45^:

3 $$nb^2 = 2l_{\mathrm{p}}\tau L$$

where *l*_p_ is the persistence length of DNA, *τ* is genomic-to-spatial distance conversion factor (nanometers per basepair) and *L* is the genomic distance separating two loci, for $l_{\mathrm{p}} \ll \tau L$. *τL* together represents the contour length along the DNA polymer separating two loci. *τ* is fit separately for each chromosome, as previous studies show their length scales differ^31^.

In a setting where multiple signals are detected for each of two genomic loci, it is ambiguous which pair of signals lies on the same chromatin fiber. This Gaussian chain model allows us to express the probability two discrete loci imaged are physically connected as a function of both its observed spatial distance and its expected spatial distance. Here, the expected spatial distance is derived from the known genomic distance separating two loci on a reference genome. In taking one step along the chromatin fiber, we can select or omit a fluorescence signal by identifying (if any) a pair of signals with unique sequence identities whose observed spatial separation is ideally congruent with its expected spatial separation. In tracing the entire chromatin fiber, the most likely polymer among imaged loci is one where the collective segment lengths along the chromatin fiber best aligns with its expected segment lengths. Our optimization objective is therefore to find the sequence of spatially resolved genomic loci that maximizes the likelihood, or CDF, of the polymer traced.

**Fig. 1:**
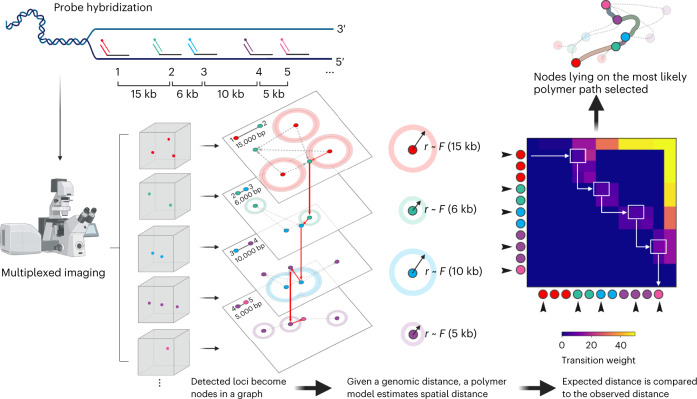
Spatial genome alignment of multiplexed DNA FISH imaging data against a reference soft-polymer structural model of DNA. Schematic of spatial genome alignment. Spatial coordinates in three dimensions (*x, y, z*) of signal detected from each imaged loci are abstracted as nodes in a graph, ordered by their appearance on the reference genome. Using a freely jointed Gaussian chain model (*F*), our aligner estimates an expected spatial distance (*r*) based on the genomic distance separating two loci. This expected spatial distance is compared to the observed distance, and an edge between two loci are connected weighted proportionally to physical likelihood. We task the aligner to find the shortest path through our adjacency matrix, which returns the sequence of spatial positions whose path length equates to the most likely polymer.

Algorithmically, we first abstract imaged chromatin fluorescent signals as nodes in a directed acyclic graph. The topological order of nodes is determined by the order of loci on the reference genome (Fig. 1). We connect each node to the adjacent nodes on the linear genome, with each directed edge emulating a polymer segment. For each directed edge, we leverage the known genomic distance separating the two imaged loci to estimate an expected spatial distance. We use both the expected spatial distance and observed spatial distance between the two imaged loci to calculate a bond probability, assigned as the edge weight. Traversing the graph from beginning to end is to find a potential chromatin fiber. Keeping track and multiplying the edge weights traversed, the score of one path reflects its physical likelihood (CDF).

Operationally, we transform the edge probabilities with a negative logarithm function into positive edge weights, such that the additive sum of edge weights reflects the polymer CDF. With this transformation, our optimization objective of maximizing likelihood is reframed as minimizing the sum of negative logarithm transformation of edge probabilities: in other words, we wish to find the shortest path through our graph representation of the polymer. Using dynamic programming, we find the shortest path through the adjacency matrix of our polymer graph not unlike traditional sequence alignment^46,47^. To account for false positives and false negative imaged spots, all valid paths are explored with the option to ‘skip’ a node permitted by a gap penalty. Since DNA loci from a chromosome must lie on the same chromatin fiber that cannot branch, finding the shortest path is to find the most probable polymer without physical discontinuity discoverable from data^48^.

**Fig. 2:**
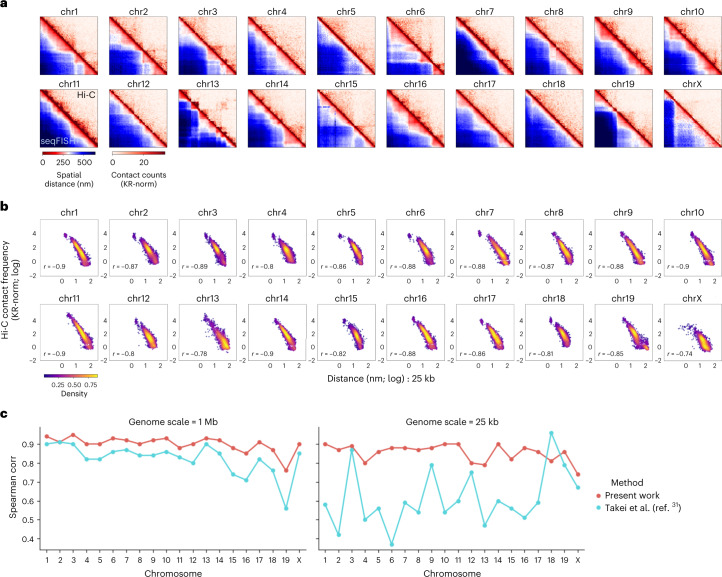
Spatial genome alignment of seqFISH+ chromatin imaging of mESCs at 25 kb and 1 Mb resolution. **a**, Heatmaps of seqFISH+ chromatin imaging of mESCs at 25 kb resolution (bottom left) juxtaposed to contact frequency from bulk proximity-ligation assay or Hi-C binned at 25 kb (top right). The distance matrix is an untabulated, median distance matrix of all single-molecule chromatin fibers identified by spatial genome alignment for every chromosome across 1,160 cells. Heatmaps for 1 Mb chromatin tracing imaging data are shown in Extended Data Fig. 4. **b**, Spearman correlation between pairwise spatial distances (*x* axis, log normalized) imaged at 25 kb resolution against Hi-C contact frequency (*y* axis, log normalized) binned at 25 kb resolution. **c**, Spatial distance to proximity-ligation correlation comparison across methodologies, at 1 Mb seqFISH+ imaging resolution as well as 25 kb resolution. For 25 kb resolution where DNA domains organize nonlinearly, we note that single-molecule chromatin fiber tracing via spatial genome alignment captures structural variations more faithfully than tabulating all pairwise spatial distances within a specified radius into an ensemble structure.

**Extended Data Fig. 1:**
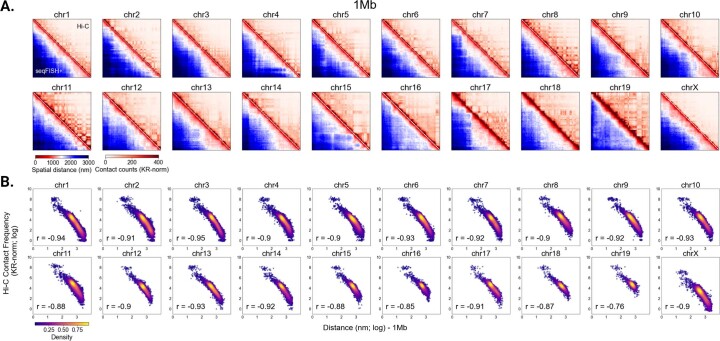
Spatial genome alignment on 1 Mb seqFISH+ chromatin imaging of mESCs. (**a**) Heatmaps of seqFISH+ chromatin imaging of mouse embryonic stem cells (mESCs) at 1 Mb kb resolution (bottom left) juxtaposed to contact frequency from bulk proximity ligation assay or Hi-C binned at 25 kb (top right), continued from Fig. 2. (**b**) Spearman correlation between pairwise spatial distances (x-axis; log-normalized) imaged at 25 kb resolution against Hi-C contact frequency (y-axis; log-normalized) binned at 1 Mb resolution.

We first benchmark our spatial genome aligner against the chromatin tracing strategy that connects adjacent genomic loci by converting tabulated distances into an ensemble contact frequency. We analyzed previously published DNA seqFISH+ genome-wide chromatin tracing on mouse embryonic stem cells (mESCs), tracing every mouse chromosome at roughly 1 Mb resolution across 1,160 single cells^31^. Whereas in published work, detected loci were binned and tabulated to convert distances into an ensemble contact frequency, our spatial genome aligner resolves single-molecule chromatin fibers at single-cell resolution across multiple genome scales. Indeed, our spatial genome aligner traces points whose median distance matrices are commensurate with KR-normalized bulk Hi-C contacts (1 Mb, Spearman correlation of −0.9 ± 0.04; 25 kb, Spearman correlation of −0.85 ± 0.04). We found our spatial genome aligner can resolve large chromatin compartments imaged at 1 Mb intervals (Extended Data Fig. 1a,b) as well as finer, single-cell chromatin domains imaged at 25 kb intervals (Fig. 2a,b). At 25-kb resolution, local chromatin structure is often nonlinearly organized into topologically associating-domain-like patterns, which may have variable boundaries, variable domain numbers and lifetimes^26–34,39–43^. Because our polymer model is a freely rotating chain of flexible segments, we found that it accommodates such abrupt changes in local topology not easily captured when tabulated in an ensemble fashion (Fig. 2a–c).

**Extended Data Fig. 2:**
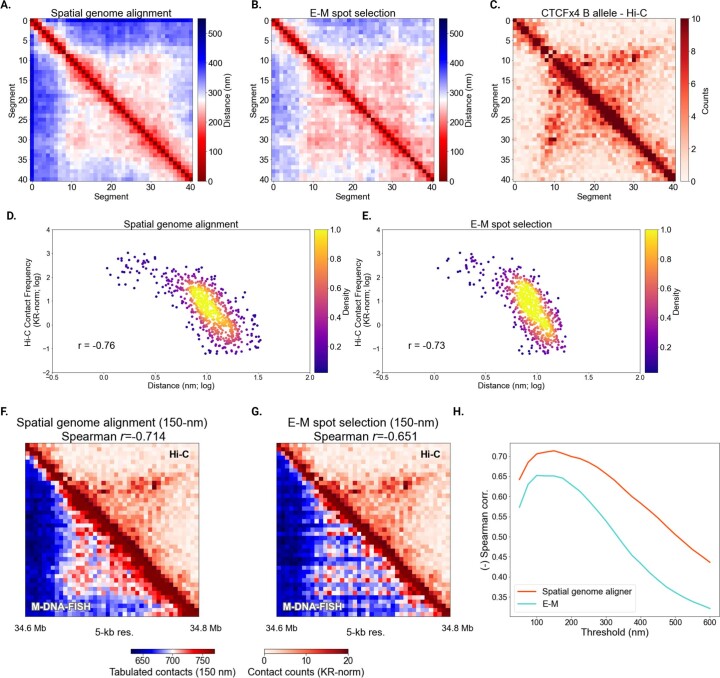
Spatial genome alignment on sequential 5 kb M-DNA-FISH imaging of mESC Sox2 locus. (**a**) Median distance matrix of spatial genome alignment resolved Sox2 locus (chr3:34601078-34811078), sequentially imaged at a finer 5 kb resolution, using a different imaging protocol based on multiplexed DNA-FISH. (**b**) Median distance matrix identified by traditional spot selection using an expectation-maximization routine previously used to analyze M-DNA-FISH datasets. (**c**) KR-normalized Hi-C contact matrix of the Sox2 locus, juxtaposed as a comparison to median distance matrices discovered by two chromatin tracing algorithms. (**d**, **e**) Correlation plot of pairwise genomic distances discovered by spatial genome alignment and an E-M spot selection routine respectively, plotted against KR normalized Hi-C contact frequency. (**f**) Heatmaps of tabulated distances (radius = 150 nm) from spatial genome alignment of M-DNA-FISH imaging (bottom left) juxtaposed to contact frequencies from Hi-C binned at 5 kb (top right). (**g**) Heatmaps of tabulated distances (radius = 150 nm) from E-M spot selection of M-DNA-FISH imaging (bottom left) juxtaposed to contact frequencies from Hi-C binned at 5 kb (top right). (**h**) Spearman correlation (y-axis) of tabulated distances from M-DNA-FISH tracing against bulk Hi-C, across a range of distance thresholds (x-axis) for tabulating distances into contacts, delineated for each of the tracing methods (spatial genome alignment and E-M spot selection).

To evaluate the performance of spatial genome alignment on finer genomic length scales, and on data from other chromatin imaging protocols, we performed spatial genome alignment on multiplexed DNA FISH data of the Sox2 locus imaged at 5-kb resolution^49^. In our previous work, we adapted a protocol based on sequential DNA-FISH^28^ to label a 210-kb genomic region on mouse chr3, spanning both the Sox2 gene locus in the F123 hybrid mESC line and its super-enhancer 110 kb downstream. By sequentially imaging these loci and tracing the chromatin, we were previously able to visualize promoter–enhancer contacts corralled within a topologically associating domain (TAD). When we applied our spatial genome aligner to this fine 5-kb resolution chromatin imaging experiment, we found our spatial genome aligner can indeed recapitulate the TAD found at this region (Extended Data Fig. 2), faithfully capturing known promoter–enhancer interactions. We note the positional uncertainties, determined using Gaussian fitting for fluorophores, can vary across imaging platforms. This uncertainty is roughly 50 nm when drift and chromatic aberration are appropriately corrected^30^.

We additionally benchmarked our spatial genome aligner with a published chromatin tracing algorithm^49^ (Extended Data Fig. 2). Previously, chromatin tracing on multiplexed DNA FISH emphasized the optical quality of a fluorescence spot, a metric incorporating (1) brightness, (2) proximity to a chromosome center and (3) relative agreement to a moving average of preceding and subsequent spots. An E–M procedure then sequentially selected one spot with the highest quality for each chromatin locus, while iteratively updating its quality scores. In contrast, our spatial genome aligner introduces another metric into spot selection—physical constraints dictated by polymer physics—as a decision criterion for selecting spots. Compared to previously published E–M spot selection algorithms (Spearman correlation −0.73), our spatial genome aligner produces a median distance matrix that achieves a similar correlation with respect to Hi-C (Spearman correlation −0.76). The difference between the two methods become larger when we tabulate distances into contacts against a range of distance thresholds, with spatial genome alignment achieving a Spearman correlation of −0.714 at a 150-nm threshold compared to the E–M algorithm’s −0.651 (Extended Data Fig. 2f–h). Notably, our spatial genome aligner performs a global optimization that incorporates the relative positioning of all imaged loci rather than a local moving average. Considering all imaged loci may be more robust against noise compared to a moving average, in the event a starting point has especially low signal-to-noise and unduly affects the downstream moving average. Additionally, our spatial genome aligner traces the most likely polymer fibers in linearithmic time using dynamic programming, compared to the heuristic E–M approach that relies on iterative convergence. Taken together, we believe that the genomic distance separating imaged loci has the potential to disambiguate spot selection and that our spatial genome aligner can be used to model chromatin fibers at multiple lengths scales, on multiple datasets, and on different multiplexed-FISH imaging modalities.

### Polymer fiber karyotyping

A nucleus may have multiple copies of a chromosome. Finding all copies has traditionally relied on identifying compact clusters of imaged loci, aggregating by chromatin fiber. A *k*-means approach of clustering assumes the ploidy of a cell is known beforehand^28,30^, and this approach is unable to accommodate copy number variations. For *k* = 2 and ploidy *n* = 1, *k*-means may inadvertently look for a nonexistent second ‘phantom’ chromosome. Conversely, for *k* = 2 and ploidy *n* = 3, *k*-means would fail to detect an entire chromosome altogether. A copy number agnostic approach of clustering, such as DBSCAN, relies on density of detected loci^31,32^, however, its density neighborhood parameter is difficult to tune. A large density neighborhood may inadvertently aggregate two spatially separable chromosomes as a single dense cluster, misassigning two separate homologs as one. A small density neighborhood may fracture an intact chromosome into separate partitions.

**Fig. 3:**
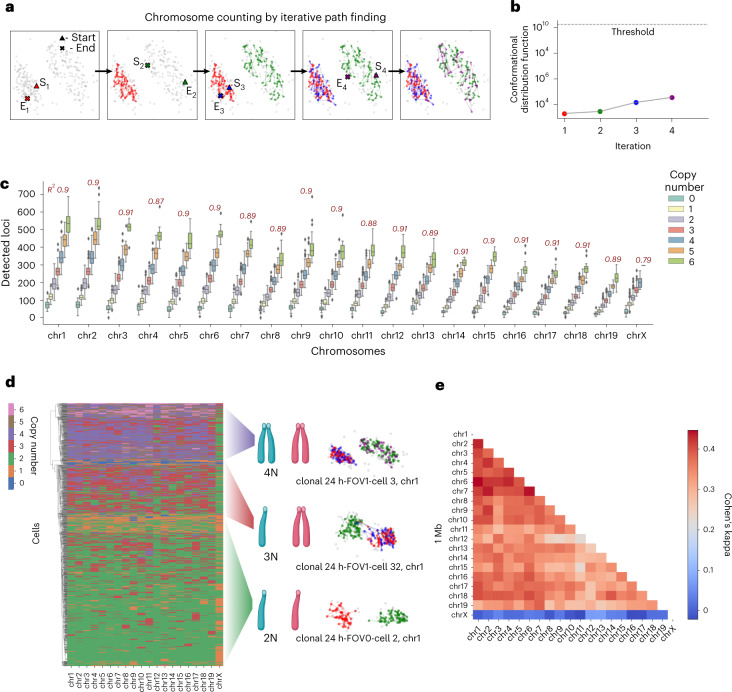
Polymer fiber karyotyping on interphase mESCs. **a**, Schematic of polymer fiber karyotyping by iterative path subtraction. For every plausible polymer found, all nodes visited on the polymer path are subtracted before a new graph is constructed for additional rounds of spatial genome alignment. Paths are extended until no physical plausible paths can be discovered. **b**, The score of each path is recorded, interpreted as the CDF of the polymer and compared to a physically unlikely threshold. Paths are extended for a given chromosome in a cell until either no more paths can be extended or a path extended has a score above threshold. **c**, Boxplot (center line, median; box, 25th to 50th percentile; bars, minima and maxima): of predicted copy number and total detected loci per chromosome (including spots omitted by spatial genome alignment) of mESC (*n* = 1,160 cells examined over four experiments). For every extra chromosome detected by polymer fiber karyotyping, we find a stepwise multiplicative increase in the total detected loci (for example, 1 chr, 100 points, 2 chr, 200 points, …). Pearson correlation coefficient evaluates this trend of detected loci per increase in assigned ploidy. **d**, Hierarchical clustering of mESCs by copy number similarity. Copy number of chromosomes is congruent across the mouse genome in a given cell with the exception of chrX in this male cell line. A dominant faction of cells is 2N in addition to a smaller faction of 3N cell with unsynchronized replication and a smaller faction of 4N cells postreplication. **e**, Heatmap of pairwise comparisons of copy number assigned by different chromosomes, with agreement scored by Cohen’s kappa.

In contrast, our spatial genome aligner provides a density or ploidy independent framework for identifying chromatin fibers. We provide all detected spatial coordinates of a chromosome and a reference genome to our spatial genome aligner, tasking it to extend (if at all possible) the most likely path from chromosome start to end. Since the path length (CDF) of a putative polymer reflects the physical likelihood of a polymer, we reasoned that the copy numbers of chromosomes inside interphase cells can be obtained simply by counting all physically likely polymer fibers. First, we set a likelihood threshold by scrambling a simulated polymer model of a reference genome such that the observed spatial distances between genes no longer abides by the genomic intervals that separate them (Fig. 3b). Next, we iteratively apply spatial genome alignment, extending polymer paths from putative seeds and subtracting nodes visited by the shortest path before searching for next shortest path, until no physically likely polymer path can be discovered (Fig. 3a). In this manner, we produce orthogonal sets of coordinates belonging to contiguous chromatin fibers with likelihood scores below our threshold. We call this process polymer fiber karyotyping.

Using chromatin tracing data spanning the mouse genome at roughly 1 Mb intervals, we performed spatial genome alignment to discover all possible chromatin fibers in the mESCs. A diploid cell should have half as many chromatin fibers as a tetraploid cell; we reasoned this should also reflect in the total number of loci detected in a cell. Comparing the total detected fluorescence signals per chromosome in a cell to its assigned ploidy determined by polymer fiber karyotyping, we notice a linear relationship. Every incremental increase in ploidy is accompanied by a stepwise, multiplicative increase in the total number of detected loci (Fig. 3c). Building on this, we compare the agreement of copy numbers assigned by each chromosome. Hierarchical clustering of copy numbers assigned by each chromosome across 1,160 cells show three distinct clusters of cells whose copy numbers are homogenously congruent for all 19 autosomal chromosomes. Namely, we see cells proportionally falling into a 6:2:2 distribution of 2N:3N:4N: cells, respectively, matching a replicative profile of highly dividing mESCs (Fig. 3d). Treating each chromosome as a separate agent for karyotyping, we quantified the copy number agreement between different chromosomes using Cohen’s kappa test. Pairwise comparisons of each chromosome against another show agreement (kappa ≥0.3), except for chromosome X (Fig. 3e). Although the spatial genome aligner had every opportunity to find as many fibers for chromosome X as it did for other autosomal chromosomes (2.618 ± 1.114 copies; mean, standard error), it found fewer copies (1.699 ± 0.669 copies) of chromosome X in this male cell line. Some heterogeneity among copy numbers between cells exists in part due to inherent imaging labeling efficiency, detection efficiency and cell segmentation challenges, as well as the stringent spatial fitting criteria of the spatial genome alignment. This gave us additional confidence that our spatial genome aligner produces accurate cell karyotypes without supervision, and that it can discriminate copy numbers in interphase where even the human eye cannot.

**Extended Data Fig. 3:**
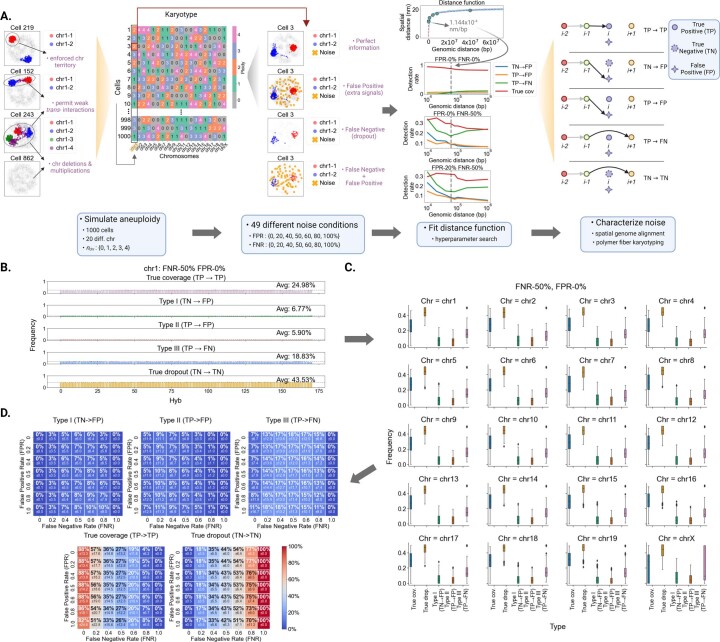
Spatial genome alignment on simulated aneuploid cells. (**a**) Schematic of simulation process generating aneuploid cells. Enforced chromosome territories segregated separate copy numbers, but permitted weak *trans*-interactions. Each aneuploid cell was subject to 49 different conditions of dropout and added noise. A power law fit to pairwise genomic-to-spatial distance allowed the estimation of expected spatial distance for a range of genomic distances (green dots, scatter plot). Subsequent alignment via polymer fiber karyotyping was parsed into 5 categories of signal selection. The best distance parameter – spatial distance divided by genomic distance – was selected based on the parameter that minimized the errors parsed, after alignment on 10 simulated cells on both perfect information (0% FNR 0% FPR) as well as imperfect information (50% FNR 0% FPR; 50% FNR 20% FPR). (**b**) Histogram of signal selection, delineated into 5 categories, for mouse chromosome 1 at 50% False Negative Rate (FNR) and 0% False Positive Rate (FPR). (**c**) Boxplot (center line – 50%tile; box – 25% to 75%tile; bar – 0 to 100%tile) of signal selection, delineated into 5 categories, for all mouse chromosomes (n = 1000 simulated cells with an average copy number of 2 per chromosome) at 50% FNR and 0% FPR. (**d**) Table of signal selection, delineated into 5 categories, summarized for all mouse chromosomes across all 49 conditions of dropout and added noise.

**Extended Data Fig. 4:**
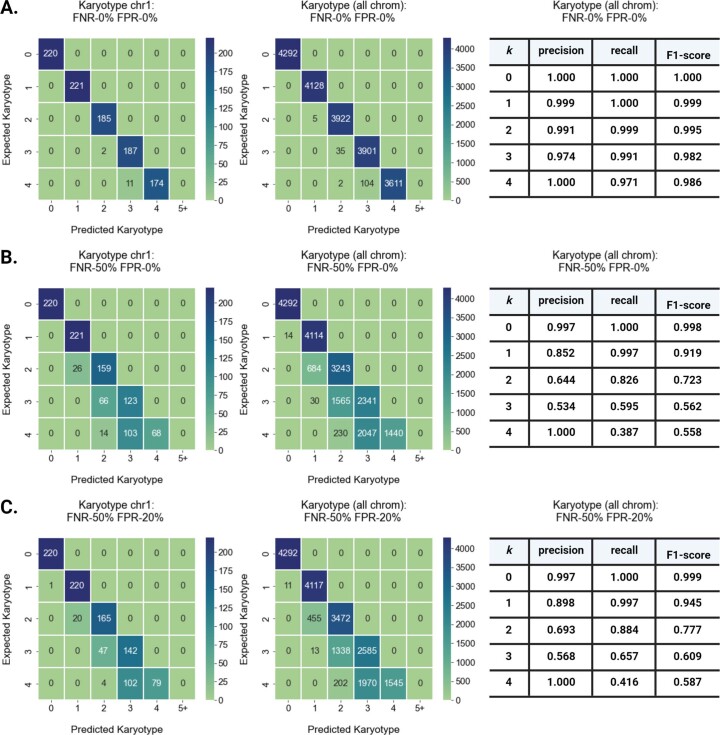
Polymer fiber karyotyping on simulated aneuploid cells. (**a**) Table of expected compared to predicted copy numbers, summarized for mouse chromosome 1 (left) and for all mouse chromosomes (middle), and its precision/recall statistics at 0% FNR 0% FPR. (**b**) Table of expected compared to predicted copy numbers, summarized for mouse chromosome 1 (left) and for all mouse chromosomes (middle), and its precision/recall statistics at 50% FNR 0% FPR. Table of expected compared to predicted copy numbers, summarized for mouse chromosome 1 (left) and for all mouse chromosomes (middle), and its precision/recall statistics at 50% FNR 20% FPR.

To assess our polymer fiber karyotyping’s accuracy in counting interphase chromosomes, we simulated 1,000 aneuploid cells. In each cell, we randomized copy numbers between 0 and 4 for each of the 20 mouse chromosomes, such that the copy number of one chromosome cannot inform the copy number of another chromosome in the same cell (Extended Data Fig. 3a). To these simulated chromosomes for which we know the true localizations of loci, we added additional layers of noise including extra stray localizations (false positive) and signal dropout (false negative) (Extended Data Fig. 3a). Surveying a grid of 49 different combinations of false positive and false negative rates (FNRs), we evaluated our polymer fiber karyotyping accuracy in the analysis of chromosome counting (Extended Data Fig. 4). We also evaluated the accuracy of spatial genome alignment in the classification of signal selection amid noise on these simulated aneuploid cells (Extended Data Fig. 3).

At the alignment level, we honed in on 50% FNR and 0% false positive rate (FPR): a condition most representative of detection efficiency and off-targets reported in DNA seqFISH+ experiments^31,32^. At this noise condition, we achieve an average true coverage rate (true positive → true positive) of 27 ± 13.2% out of a maximum 50% true positive loci (Extended Data Fig. 3d). In fact, even in the setting of 0% FNR and 0% FPR (perfect information) our aligner captures 88 ± 13.2% true coverage rate out of a maximum 100% true positive loci (Extended Data Fig. 3d). This strongly suggests our aligner’s requirement to fit spatial distances between loci to be congruent with its genomic distance is extremely stringent. Indeed, the most dominant error made by our aligner is the omission of true positives or Type III error true positive → false negative), across all chromosomes (Extended Data Fig. 3c) and across all noise conditions (Extended Data Fig. 3d). At 50% FNR 0% FPR our aligner averages a Type III (true positive → false negative) error of 18 ± 13.2% across all chromosomes (Extended Data Fig. 3d). In comparison, we report an average 6 ± 6% Type I error rate (true negative → false positive), where we select a false positive spot in the absence of true signal (Extended Data Fig. 3d). This again suggests our aligner is making highly specific judgments in spot selection. The lowest source of error is a type II error (true positive → false positive), where the selection of a false positives in the presence of true signal hovers around 5 ± 4.8% at 50% (Extended Data Fig. 3d). Since we set an upper limit for number of loci skipped, the omission of true positive loci may accumulate for especially high signal dropout conditions, leading to early termination of alignment and potentially missed chromosomes.

At the karyotyping level, we found our routine achieves near perfect precision recall in the setting of perfect information (Extended Data Fig. 4a) but begins to miss chromosomes at increasing dropout rates (Extended Data Fig. 4b). We wondered whether our aligner ever ‘alternates’ between loci, leaving behind enough ‘residual points’ that lead to an overcounting of chromosomes on second iteration. Instead, we found our algorithm may undercount but almost never overcount, across a range of noise conditions (Extended Data Fig. 4a–c). Even when we added extra noise signals, our algorithm did not mistake these stray localizations for additional chromatin fibers (Extended Data Fig. 4c). We found that chromosome detection efficiency slightly increases with added noise (Extended Data Fig. 4c). For instance, at 50% FNR 0% FPR, our polymer fiber karyotyping identifies 3,243 of 3,927 2N chromosomes. At 50% FNR 20% FPR, our routine identifies 3,472 of 3,927 2N chromosomes. We attribute this improved chromosome detection sensitivity to a ‘placeholding’ effect that stems from the design of our aligner. Just as the aligner has an upper limit to total number of loci skipped per chromatin fiber, our aligner also has an upper limit to the number of loci skipped at any given locus. Dropout without any noise may result in consecutive missing signals stretching beyond the furthest allowable skip. Yet in the setting of signal dropout and added noise, our algorithm may potentially align to a placeholding ‘noise’ spot, taking the penalty of an incongruent spatial segment to complete the alignment from chromosome start to end.

### Aggregation of homologous chromosomes in tetraploid mESC cells

**Fig. 4:**
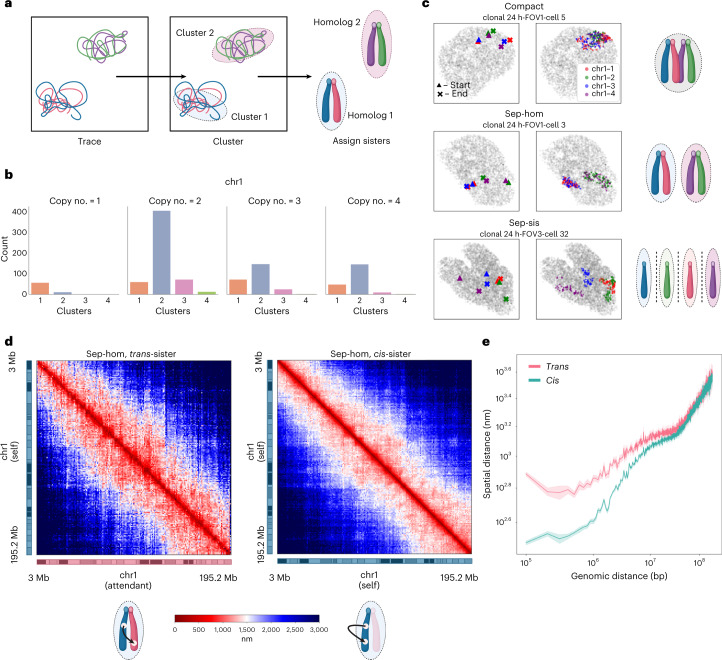
Sister chromatid interactions in mESCs resolved by polymer fiber karyotyping. **a**, Schematic of homolog assignment. Spatially separable structures are clustered using DBSCAN and polymer fibers residing in the same spatial neighborhood are classified as sisters of the same homolog. **b**, Histogram profile of chr1 traced at 1 Mb resolution across 1N, 2N, 3N and 4N cells (*n* = 1,160 cells over four experiments). **c**, Representative classes of homologous chromosome organization in 4N mESC cells. A dominant fraction of cells has two spatially separable homologs (sep-hom) residing in chromosome territories, while another fraction of cells has one spatial cluster wherein both pairs of sisters from different homologs interact (compact). A marginal population has three or more spatially separable structures showing separated sister chromatids (sep-sis). **d**, Median distance matrices of intra-sister chromatin fiber versus inter-sister chromatin fiber interactions showing a tight locus pairing between sister chromatids of the same homolog. **e**, Spatial distance separation (bolded line, mean; shaded band, 95% confidence interval) across genomic length scales between loci on the same sister chromatin fiber (*cis*) versus separation between loci across different sister chromatin fibers (*trans*).

Of the putative 4N chromosomes predicted by our spatial genome aligner, we asked whether these are polyploid cells with four separable chromosomes before replication, or diploid cells with two pairs of sister chromatids after replication (Fig. 4a–c). As sister chromatids are shown to be tightly paired in a parallel fashion^50–53^, we reasoned that if two chromatin fibers reside in the same spatial neighborhood, they are likely sister chromatids of the same homolog. We performed density-based clustering to assign fibers of every ploidy to homologs. Under a set density parameter, most diploid cells had two spatially resolvable fibers singularly residing in different territories (Fig. 4b). Notably, under the same density parameter most tetraploid cells also had not four spatially resolvable fibers, but also two clusters of paired fibers that cannot be parsed by eye or by known clustering algorithms (Fig. 4b).

To test whether these paired fibers are indeed sister chromatids, we examined the *trans-*fiber loci distances relative to the *cis-*fiber loci distances. In agreement with published sister chromatid sensitive Hi-C on *Drosophila* and human cell lines^52,53^, paired fibers in mESCs resolved by our spatial genome aligner are spatially coupled. Explicitly, a given locus of one sister chromatid is followed by the same locus on its attendant sister chromatid, faithfully ‘shadowing’ each other (for example, chromasome 1 (chr1) *µ* = 1,606.8 nm separation; 95% CI (1,540.3, 1,734.5)) (Fig. 4d). Congruent with previously published work, the spatial distance between *cis-*fiber interactions is closer for smaller genomic distances but which converges with *trans-*fiber interactions above 10 Mb (Fig. 4e). Given the parallel nature of pairing and recapitulation of sister chromatid interactions found by sister chromatid sensitive Hi-C, we presumed the tetraploid cells were in fact replicated diploid cells showing paired sister chromatids.

**Extended Data Fig. 5:**
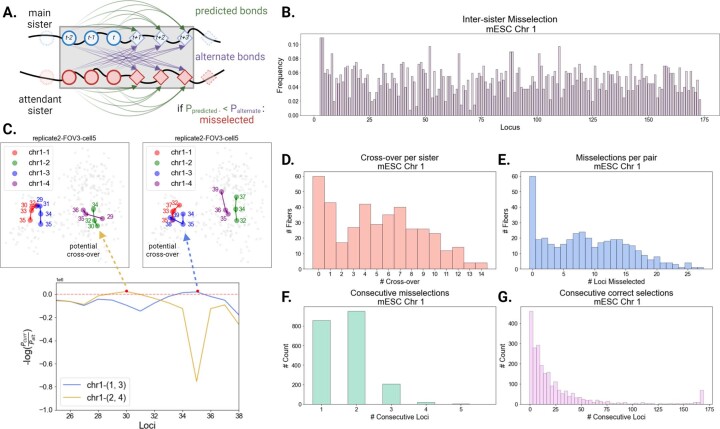
Assessment of misselection between tightly coupled sister chromatids. (**a**) Schematic of misselection assessment. For every tightly paired sister chromatid, a sliding window of three downstream loci are allowed to exchange with loci on the other sister. Bond probabilities between three upstream loci and three downstream loci are calculated. Original bond probabilities are compared to the cross-over probabilities to evaluate misselection. (**b**) Bar-plot of misselection frequency per locus on mESC chr 1. (**c**) Representative image of loci belonging to sister chromatids whose bond probabilities are more probable (-log(P_predicted_/P_alternate_) > 0) if loci are exchanged between sisters. (**d**) Number of cross-over events, defined as sequences of misselections of any length, per sister chromatid. (**e**) Number of misselected loci per sister chromatid. (**f**) Histogram of length of consecutive misselections across tightly paired sister chromatids. (**g**). Histogram of length of consecutive correct selections across tightly paired sister chromatids.

**Extended Data Fig. 6:**
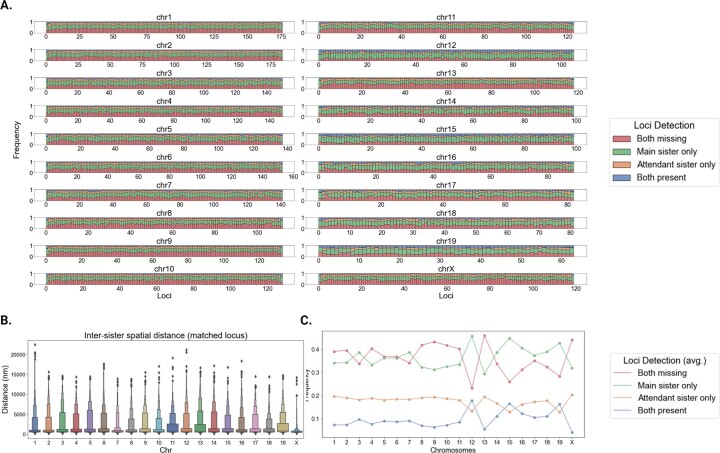
Spatial genome alignment locus detection in putative sister chromatids. (**a**) Frequency of loci detection on tightly coupled sister chromatids, stratified by detection on both sister chromatids, detection on main sister, detection on attendant sister, and failure of detection on either, for each of the mouse chromosomes. (**b**) Boxen plot (center line – 50%tile) of inter-sister spatial distance, for each matched locus, across all mouse chromosomes (n = 1160 cells). (**c**) Detection rate averaged across all loci of a given chromosome, for each chromosome, stratified by detection on both sister chromatids, detection on main sister, detection on attendant sister, and failure of detection on either, for each of the mouse chromosomes.

**Extended Data Fig. 7:**
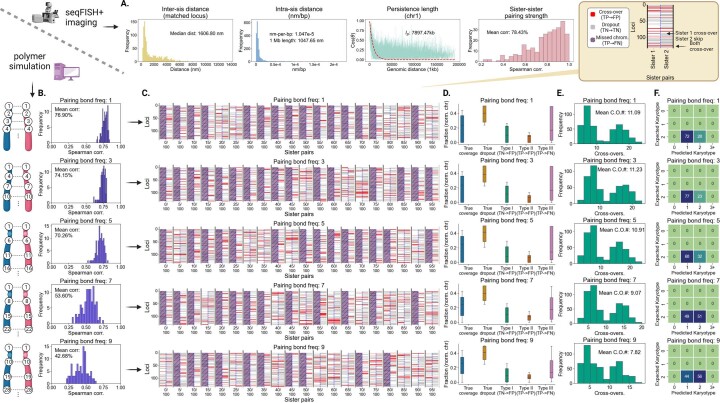
Spatial genome alignment on simulated paired chromatin fibers. (**a**) Summary of inter-sister locus distance, intra-sister locus distance, persistence length, and sister-chromatid pairing strength for DNA seqFISH+ imaging of mouse chromosome 1. (**b**) Spearman correlation plot of all 100 pairs of paired fibers, delineated by the frequency of pairing bonds. (**c**) Representative heatmap of twenty simulated pairs at different pairing strengths, traced via spatial genome alignment. Red delineates misselected loci (cross-over), while gray delineate loci dropped out as input to emulate 50% FNR 0% FPR. (**d**) Bar plot (center line – 50%tile; box – 25 to 50%tile; bar – 0 to 100%tile) of locus selection summarized for alignment on simulated pairs of different pairing strengths (n = 100 pairs of fibers, per pairing strength), parsed into 5 categories of locus selection. (**e**) Frequency of cross-over during spatial genome alignment on simulated chromatin pairs of different pairing strengths. (**f**) Table of expected compared to predicted copy numbers for polymer fiber karyotyping on simulated chromatin pairs of different pairing strengths.

Because these sister chromatids are tightly coupled, it is plausible that loci belonging to one sister are inadvertently selected by its attendant sister and vice versa. To assess this misselection event, we revisited all putative sister chromatid pairs of mESC chr1. Examining a sliding window of three loci, we allowed sisters to exchange their selected spatial positions for these three loci. Next, we re-evaluated the resultant bond probabilities between three upstream loci to the three downstream exchanged loci (Extended Data Fig. 5a). If the new bond probabilities are more probable than the original on both sister chromatid fibers, we call this a misselection event (Extended Data Fig. 5b). A sequence of consecutive misselections of any length is called a cross-over event (Extended Data Fig. 5d). We found that our spatial genome aligner is susceptible to these errors when tracing paired sister chromatids, and has an average locus misselection rate of 5.06 ± 1.84%. The mean cross-over per fiber is 5.08 ± 3.74 (Extended Data Fig. 5d). One source of misselections lies in the sparsity of data (Extended Data Fig. 6a,c). Rarely are two signals simultaneously detected for a locus (chr1, 7.34%) and present for selection by both the main sister and its attendant (Extended Data Fig. 6a,c). In fact, for chr1 34.5% of all cases with zero signals are detected for a given locus. In the absence of robust signal, our polymer fiber karyotyping routine is greedy. Whichever sister is discovered first is incentivized to select as many detected loci as it sees fit, at the cost of potentially selecting signals that belong to the other sister. A signal cannot be re-selected by subsequent sisters. When only one signal is detected for a given locus, we therefore see a disproportionate imbalance of the single locus signal assigned to the main sister traced first (34%) compared to the signal assigned to the attendant sister traced second (19.6%). In a controlled simulation of paired sister chromatid fibers, we find that the predominant cross-over error our aligner makes is a cross-over in the absence of signal on the present sister chromatid but presence of signal on the attendant sister (type I, true negative → false positive; Extended Data Fig. 7d). In the presence of signal on both sister chromatids, our aligner rarely makes cross-over errors (Type II; true positive → false positive). These results hold true for a range of sister chromatid pairing strengths (Extended Data Fig. 7b,d). Future improvements in experimental detection efficiency, sister chromatid specific labeling and algorithmic flexibility permitting revisiting signals with higher intensity (indicative of coincident signals) may decrease this misselection error and improve sister chromatid tracing coverage.

Canonically, homologous chromosomes are divorced from each other in the nucleus and are widely acknowledged to reside in separate territories^6^. Yet, of the 207 cells tetraploid for chr1 we find two predominant patterns: three-quarters (146 out of 207 cells) bearing two spatially separable clusters presumed to be different homologs (sep-hom) and, strangely, a quarter (49 out of 207 cells) with all four fibers are coalesced (compact) (Fig. 4c). We note a marginal population of cells (12 out of 207 cells) with three or more separable structures corresponding to separated sister chromatids (sep-sis). In case our clustering density parameter had inadvertently grouped two separable homologs together, we visually inspected each putative compact 4N chr1. To our surprise, we find that the majority of the compact state (31 out of 49 candidates) cells, cumulatively 2.67% of total cell population, has four chromatin fibers spatially intermingling and which cannot be separated by eye.

Why would the newly replicated homologous chromosomes coalesce? While homologous chromosome generally lie in separate chromosome territories^54,55^, some species such as *Drosophila* exhibit extensive homologous pairing in somatic cells^56^. It is thought that communication between homologs, across a number of species^55–57^, underlies gene regulation^58^, chromosome counting^59^, DNA repair (with resultant loss of heterozygosity)^60–63^, and even chromosome topology^64^. The *trans*-homologous interactions identified in mammalian cells under physiological conditions have been sparse^65,66^. However, there have been reports of such events in the setting of human diseases^67,68^, suggesting this rarity of homologous pairing may be either species specific or due to mechanisms in mammals that deliberately prevent such interactions^55^. Here we suggest the possibility that multiplexed DNA FISH and chromatin tracing may help better understand this potential for *trans*-homologous interactions.

**Fig. 5:**
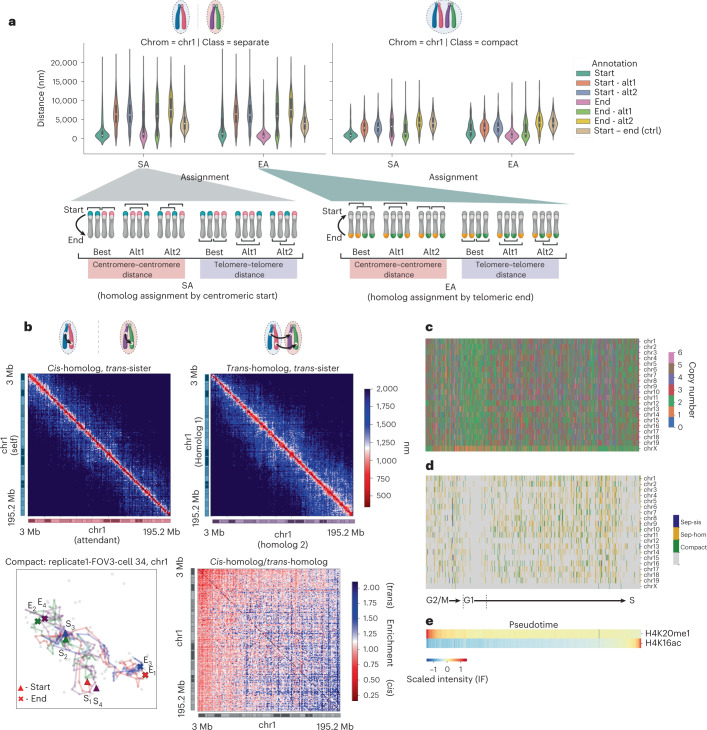
Sister chromatid interactions in compact homolog state. **a**, Violin plot (center dot, median; gray bar, quartiles) comparing spatial separation between centromeric starts or between telomeric ends of sister chromatids, stratified by sister chromatids in separate chromosome territories (*n* = 146 cells across four experiments) and by sister chromatids in a compact territory (*n* = 49 cells across four experiments). All six permutations, including sisters assigned by closest centromeric starts (SA) and its alternate pairings are shown, as well as sisters assigned by closest telomeric ends (EA) and its alternate pairings. Note that in mouse chromosomes, the centromere is located at the end of the chromosome. We refer to the centromere and centromeric to delineate the centromeric telomere; the telomere and telomeric to delineate the noncetromeric mouse telomere. The pairwise distance between a centromeric start and its telomeric end on the same fiber is presented as a control for unpaired loci. The colors of the chromosome cartoons do not indicate phased labeling, and instead emphasize combinations of grouping two fibers. **b**, Median distance matrix of *cis-*homolog *trans-*sister chromatids versus *trans-*homolog *trans-*sister chromatids. A representative structure is shown on the bottom left, with a heatmap showing *cis-*homolog to *trans-*homolog distance ratio on the bottom right. **c**–**e**, A heatmap of assigned copy numbers (**c**) and tetraploid chromosome organization state (**d**) ordered along pseudotime determined as a function of cell-cycle markers H4K20me1 and H4K16ac (**e**). Chromosome images in **a** and **b** created with BioRender.com.

To understand our observed compact chromosome territories, we began by parsing which two fibers belong to one homolog within the compact structure. Spatial proximity prohibits clustering from separating homologs and assigning pairs of fibers as sisters. Since true sister pairings should involve two fibers shadowing each other, we reasoned sisters can be assigned by proximity of a given locus on two fibers. There are two natural assignments: grouping the closest pairs by the starting locus (SA; mouse centromere, which, in mouse, is located at the end of each chromosome), and grouping the closest pairs by the end locus (mouse telomere, noncentromeric mouse telomere) (Fig. 5a). While both are telomeres, we use centromeric and telomeric to denote locations: namely the start and end locus of the chromosomes, respectively. We note spatial proximity may cause our spatial genome aligner to inadvertently select spots belonging to other fibers. Therefore, we explored the centromere–centromere distances as well as telomere–telomere distances of the two remaining permutations. Specifically, these permutations correspond to the best possible alternate pairing (alt1) as well as the remaining pairing (alt2), ranked in this order.

When replicated homologs reside in separate chromosome territories, we find that the telomeres of putative sister chromatids grouped by their centromere are likely coupled (Fig. 5d, left). The mean distance separating telomeres of centromere sisters (mean 3,857.5 nm; 90% CI (3,451.5, 4,263.5)) is smaller than the distance between its centromere–telomere and not known to interact (mean 4,474.9 nm; 90% CI (4,295.8, 4,654.1)). In contrast, the next best alternative pairing has a telomere separation (mean 5,767.4 nm; 90% CI (5,342.2, 6,192.6)), larger than two noninteracting loci. In the same manner, the centromeres of putative sisters grouped by their telomere (end locus) are also tightly coupled. All other alternate pairings exhibit a spatial separation above that of two noninteracting loci.

When replicated homologs coalesce, we find that putative sisters grouped by their centromere may lose pairing at their telomeres. The mean distance separating telomeres of Start Assigned (SA) sisters is similar (*µ* = 3,286.5 nm; 90% CI (2,835.9, 3,737.1)), compared to the distance between two noninteracting loci (*µ* = 4,310.9 nm; 90% CI (4,110.2, 4,511.7)). The next best alternative pairing has a telomere separation (*µ* = 2,544.2 nm; 90% CI (2,131.2, 2,597.1)) closer than the centromere assigned telomere distance. Should this be due to misassignment, then all three pairing scenarios should share a uniformly unpaired distance distribution with mean distances above coupling. Yet, there almost always exists an alternate pairing between putative homologs that theoretically should not interact. The same analysis on End Assigned (EA) sisters confers an ambiguous result, likely due to this loss of pairing.

The tendency for *cis-*homolog coupling decreases moving away from the centromere, resulting in a ‘flare-up’ of putative *trans-*homolog interactions near the telomere of chromosomes (Fig. 5b). Since DNA seqFISH+ labels discrete genomic loci, we are not able to visualize the contiguous polymer physically linking imaged loci. Additionally, DNA seqFISH+ probes do not discriminate homologs. Our reanalysis of misselection events (Extended Data Fig. 5) and analysis of simulated sister chromatid pairs (Extended Data Fig. 7) suggest our aligner may inadvertently cross-over between loci that belong to other chromatin fibers, albeit at lower rates than true coverage. We are therefore unable to definitively show all spots traced by our aligner lie on the same contiguous fiber, and that our assignments by loci proximity are always sisters of the same homolog. However, our spatial alignment analysis in bulk suggests a possible loss of sister pairing toward the telomeric end and increased *trans-*homologous interaction within this compact 4N state. We ordered the cells along the same pseudotime axis as determined using previously published cell-cycle markers (H4K20me1, H4K16ac) (Fig. 5c–e). We found that this compact 4N state is scattered throughout interphase leading up to S phase. Additionally, this compact 4N state is rarely synchronized across multiple chromosomes, and appears to occur stochastically. This suggests *trans-*homologous interactions between replicated chromosomes are likely uncoordinated and not initiated by a particular cell-cycle checkpoint.

### Extranumerary paired chromosomes in mouse cortical neurons

Extranumerary chromosomes and more broadly aneuploidy have previously been reported in the brain, by both FISH and single-cell sequencing^69–73^. This copy number variation is thought to underlie a functional diversity adequately supporting neural complexity^70^. In DNA seqFISH+ imaging of mouse cortex, we sought to evaluate such copy number variations with polymer fiber karyotyping. Because cryosectioning may cut through nuclei, leaving some chromosomes (in whole or in part) out of view, we analyzed 701 fully segmented mouse neurons^32^, all lying in the center *z*-sections of female mouse cortexes derived from three biological replicates. Of these intact nuclei, we honed in on excitatory neurons: the predominant cell type in this dataset (*n* = 458/701 neurons).

**Fig. 6:**
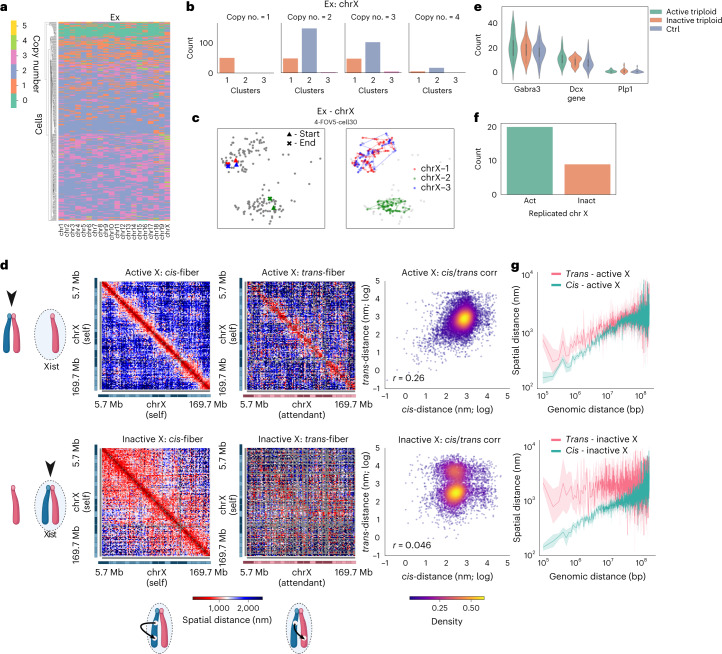
Copy number variations in mouse cortical neurons. **a**, Hierarchical clustering of *n* = 458 excitatory cortical neurons from female mice by copy number similarity. **b**, Histogram profile of chromosome X traced at 1 Mb resolution across excitatory cortical neurons bearing 1, 2, 3 and 4 copies. **c**, Representative image of 3N chromosome X organization in excitatory cortical neurons. Two chromosome territories are preserved despite harboring three chromatin fibers; two fibers reside in one territory while the third is divorced in a separate territory. **d**, Median distance matrices comparing *cis-*fiber loci distances to *trans-*fiber loci distances on the active and inactive chromosome X. The log normalized distance density heatmap (right) examines the correlation between *cis*–*trans* distances, on the active and inactive X chromosome. **e**, Violin plot (center dot, median; gray bar, quartiles) of gene expression counts of three genes lying on chromosome X, comparing the effect of gene dosage on transcription, subclassified by chrX activation status (active, *n* = 20 cells across four experiments; inactive, *n* = 9 cells across four experiments; control: *n* = 20 randomly selected cells across four experiments). **f**, Count plot of excitatory cortical neuron nuclei with three copies of chromosome X, classified by the activation status of the territory where two of the three chromosome X fibers colocalize. **g**, Spatial distance separation (bolded line, mean; shaded band, 95% confidence interval) across genomic length scales between loci on the same chromatin fiber (*cis*) versus separation between loci across separate chromatin fibers (*trans*), subclassified by chromosome X activation status.

Our karyotyping routine reports 58.05 ± 6.38% (mean, standard deviation) of a given chromosome as 2N. It classifies 13.82 ± 3.03% of a given chromosome as 1N, and 19.68 ± 5.08 of a given chromosome as 3N (Fig. 6a). Unlike cell line imaging, karyotyping on multiplexed-FISH imaging of tissue is challenged by (1) poor probe labeling, (2) higher background fluorescence, (3) difficult cell segmentation and (4) aforementioned cyrosectioning artifact that may collectively contribute to missed chromosomes (Fig. 6a). Additionally, our extensive analysis of polymer fiber karyotyping on simulated aneuploid cells indicates that, on data with low imaging detection efficiency, our aligner may potentially undercount chromosomes (Extended Data Fig. 4). Our aligner’s criteria of fitting the spatial separation between each pair of loci to be congruent with its genomic distance is extremely specific in filtering out noise, sometimes at the cost of lost sensitivity. It currently has a set threshold for total number of skipped loci that qualify a valid polymer. It also has a restriction in the number of loci can be skipped at a step in spatial genome alignment. Either successive dropout in a region of a genome (despite all other loci fully detected) or cumulative dropout across the chromosome may terminate our alignment, potentially missing a chromosome. In this mouse brain imaging dataset, the detection efficiency hovers around 47.3 ± 16.2% (2,326 ± 795 spots; 2,460 labeled loci) for 1 Mb tracing^32^. At this sparsity, we therefore believe the number of chromosomes classified as 1N is at least partially contributed by a tendency to undercount in our algorithm. The potential to undercount, however, led us to take a closer look at extranumerary chromosomes (3N) in cortical neurons.

**Extended Data Fig. 8:**
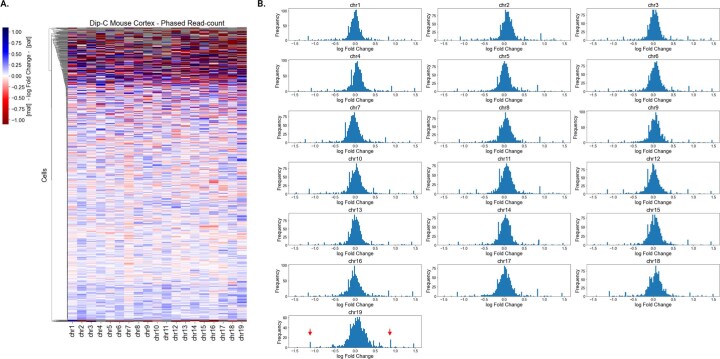
Haplotype-resolved single-cell sequencing of mouse cortical excitatory neurons. (**a**) Heatmap of log2 fold change of haplotyped Dip-C reads assigned to the paternal vs maternal allele. (**b**) Histogram of log2 fold change of haplotyped Dip-C reads. Most reads are balanced between the two haplotypes, while a distinct peak around 1 and −1 (red arrows) are present for every chromosome.

Due to data sparsity, many loci are absent on putative extranumerary chromosomes. To ascertain these are indeed three separate chromatin fibers and not two fibers parsed into three, we filtered all putative 3N chromosomes seeking fibers that are routed through three separate detections per locus for at least three different loci. We note that each detection of a locus under DNA seqFISH+ imaging is not a one-time detection, but in fact a temporal barcode wherein a locus is detected at least four out of five times, at the correct temporal sequence, while satisfying error correction. We also note that in our simulated aneuploid cells and in simulated sister chromatid pairs, despite high signal dropout and simulated noise, our aligner does not split one chromatin fiber into two (Extended Data Fig. 7f). Even still, we find that 8.72 ± 4.18% of each chromosome are predicted to be 3N under this more stringent criterion. We validated our karyotyping results by analyzing haplotype-resolved single-cell Dip-C sequencing on mouse cortical neurons classified as excitatory^74^ (Extended Data Fig. 8a). By counting total reads, as well as inspecting the relative fold change of reads assigned to the maternal versus paternal haplotype, most sequenced neurons have balanced haplotyped reads (|log_2_| fold change <0.9). Also, 10.82 ± 1.31% of every chromosome have twice (|log_2_| fold change ≥0.9) as many reads of one haplotype as the other (Extended Data Fig. 8a,b). Not only may this reflect copy number variations prevalent in cortical neurons, it may also indicate the extranumerary chromosome is potentially contributed to by one haplotype.

**Extended Data Fig. 9:**
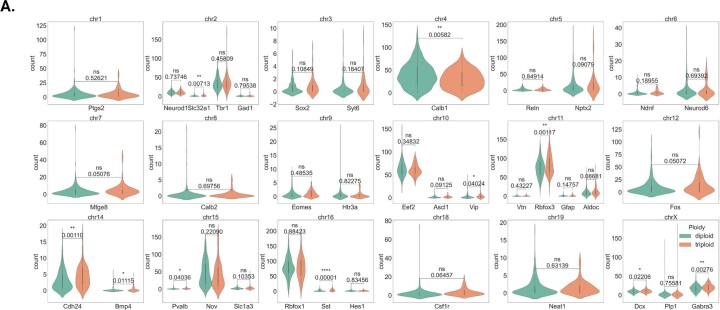
Effect of gene dosage on transcription in excitatory mouse cortical neurons. (**a**) Violin-plot (center dot – median; gray bar – quartiles) of RNA transcripts detected by seqFISH+ RNA imaging, corresponding to genes traced by seqFISH+ DNA imaging (excitatory neurons - n = 458 cells across 3 experiments). RNA counts are stratified by gene dosage, delineated by mouse chromosome. Statistical significance and p-values evaluated by two-sample pairwise t-tests are labeled above, comparing 2N vs 3N nuclei of a given chromosome.

Because single-cell sequencing affords relative copy numbers of reads and fails to capture the nuclear organization of the aneuploid cells, we inspected the spatial organization of these copy number variations. Looking at chromosome X, where the inactive chromosome is distinguished from the active by RNA imaging of *Xist*, density-based clustering reveals that excitatory neurons with three chromosome X have predominantly two chromosome territories (Fig. 6b). One chromatin fiber is standalone, while the two remaining fibers are constituents of the same territory (Fig. 6c). Of the doubly occupied chromosome territories, two-thirds are devoid of any *Xist* signal, suggesting a preference for active chromosome X (*P* = 0.049369, one-sided binomial test; Fig. 6f). Despite different gene dosage, we detect no significant gene expression relative to cells with two detected copies of the X chromosome, irrespective of chromosome X activation status (Fig. 6e). Most labeled genes show no significant change relative to elevated gene dosage (Extended Data Fig. 9).

Between fibers in the doubly occupied chromosome territory, the active chromosome X fibers show some degree of pairing (Spearman *r* = 0.26) at a locus-to-locus level with its attendant fiber (Fig. 6d,g). This manifests as a strong diagonal in the *trans-*fiber pairwise distance matrix, as well as a spatial distance distribution mimicking that of *cis*-fiber pairwise distances. The same cannot be said of the inactive chromosome X, whose double constituents bear little resemblance (Spearman *r* = 0.046).

## Discussion

Here we present a spatial genome aligner for multiplexed DNA FISH data. We show that this framework resolves chromatin fibers from discretely labeled positions of genomic loci, amid noise and signal dropout. In our spatial genome aligner, each observed locus’ spatial position is checked against a reference model of a polymer chain. This reference model, a Gaussian chain abstracting connections between imaged loci as bond probabilities, dictate that even a highly variable structure as DNA follows predictable patterns of distance separation between loci. Our model uses a fixed scaling factor to roughly estimate spatial distance from genomic distance. We recognize that DNA compaction is known to change throughout the genome, and our fixed scaling factor does not recapitulate the exact spatial distance separating each locus imaged. However, we hypothesized this estimated distance can inform soft decisions in the selection of imaged DNA loci, in addition to considering optical qualities of signals detected, to trace chromatin structures. We found this framework captures chromatin compartments and domains also found in Hi-C, on multiple lengths scales and across different chromosomes. Future frameworks may fuse other channels of information, such as A/B compartments, chromatin modifications, gene expression levels and association with nuclear structures to estimate different distance parameters throughout the genome during chromatin tracing.

Our algorithm falls into an early lineage of spatial genome aligners, including work by Ross et al. that abstracts connections between loci as polymer segments and whose edge weights are proportional to physical likelihood^45^. Chiefly, whereas a reference polymer structure can reconcile each individual locus’ most likely spatial position using the forward–backward algorithm^45,48^, we demonstrate the use of dynamic programming to find the most plausible sequence of spatial positions discoverable^48^. In other words, finding the shortest path in our graph representation is to find the most physically likely polymer without any physical discontinuity. Our aligner builds connections between loci in the sequential order they appear on the reference genome. Currently, this prevents discovery of certain structural variants, such as inversions, translocations and duplications. However, we believe finding the most likely contiguous polymer may be instrumental to uncovering copy number variations at the single-cell level. Through iterative subtraction of shortest paths to find all valid polymers, our data indicate a capacity to recover sister chromatids otherwise mistakenly grouped as one chromosome fiber. We therefore propose a new form of karyotyping called polymer fiber karyotyping that is density or clustering independent. Ascribing a physical likelihood to polymers allows us to estimate the copy number of chromosomes, paving way for the study of copy number variations in interphase for which the expected copy number is unknown. For instance, the study of oncogene amplification in the setting of cancer heterogeneity is currently limited by reliance on compact alignment of probes in metaphase spreads^22^. It is also held back by uncertainty in measurement due to unknown true copy number postoncogene amplification.

Perhaps not unexpectedly, a potential to resolve discrete polymer fibers instead of tabulating chromosome positions can point to inter-chromosomal interactions. Multiplexed-FISH captures in high throughput native chromosome structures directly in intact nucleus among a range of different replicative states. Chromosomes undergo transformative structural change throughout the cell cycle, disassembling the interphase nucleus to condense into sister chromatids during mitosis: a process that has been historically studied using proximity-ligation sequencing^1^. And yet, so far, sister chromatid level interactions have been difficult to resolve from imaging. This is perhaps due to a lack of biochemical labeling that can discriminately label sisters that are identical in sequence. Here, we propose a computational model relying on statistical mechanics to resolve sister chromatid interactions from fluorescence imaging and although our spatial genome aligner awaits further field testing, it has enabled us to model sister chromatids from several imaged loci difficult to discern by the human eye. While most replicated homologs seem with our method to be divorced and reside in separate territories, our modeling suggests compact territories where all four sister chromatids of a given chromosome spatially aggregate. Whereas in separate territories each sister seems to be shadowed by its attendant sister, we believe that in the compact territory sister chromatids can lose sister pairing and might even pair with the other homolog. Such an interpretation is evocative of a cross-over event, which is thought to occur in mitotic cells at exceedingly rare frequencies (roughly 1–2%) consistent with our observation^60,61^. This deserves further investigation to study whether genetic information is indeed being exchanged and may bring compelling insight to the regulatory mechanisms of loss of heterozygosity underlying disease. Mechanistically, how a sister chromatid eschews pairing with its own homologous attendant and assiduously choose a sister from the other homolog is a mystery. We acknowledge that diffraction-limited widefield microscopy may ‘merge’ two signals belonging to separate chromatin fibers. Multiplexed STORM (that is, Oligo-STORM)^25,27^, which can precisely resolve chromatin organization with single-molecule sensitivity at subdiffraction limit resolution, may provide complementary information on sister chromatid pairing and global organization. Additionally, as homologous interactions are highly species specific (that is, Diptera)^55–58^ we believe this study would benefit from multiplexed DNA FISH in multiple model systems to study the permeance of this phenomenon. Studies such as these will simultaneously test the robustness of our spatial genome aligner. For example, to what extent might the inferences of copy numbers and sisters by the method reflect artifacts arising from suboptimal primary imaging data? Does the size of each targeted region, which if too large may on imaging disperse into multiple spots, affect our results? Will open, actively transcribed regions exacerbate this potential? Such questions regarding our approach could be addressed through the application of our approach to various biological systems across many laboratories.

Multiplexed DNA FISH affords an intimate look into the elusive inner realities of genomic mosaicism in the brain. We chronicle the intranuclear spatial organization of copy number variations, with single-cell sensitivity and at whole-genome scale, heretofore only reported as frequencies^69–73^. We show that in neurons with three copies of a chromosome, the extranumerary chromosome shares a chromosome territory with another chromatin fiber, preserving two chromosome territories in the nuclei. Within the doubly occupied territory, each chromatin fiber appears to shadow each other, evocative of sister chromatids previously imaged in dividing mESCs. In our study, we focus on the X chromosome, previously annotated in this dataset by the detection or absence of *Xist* RNA signal. We note that the absence of *Xist* signal as a proxy for identifying the active X chromosome may not unequivocally identify the active X chromosome. We also cannot rule out the possibility double occupancy itself induces loss of *Xist* expression, or expression of genes on other chromosomes, potentially explaining why we detect no gene dosage effect across copy number variations (Extended Data Fig. 9). Further, we cannot exclude the possibility that active state influences probe labeling efficiency and imaging detection errors. The role and origin of the extranumerary chromosome is unclear. One possibility is that the extranumerary chromosome is the derivative of a nondisjunction event, occurring in a neuroprogenitor during development. Why these chromatin fibers did not dissociate postdivision is unclear. Another possibility is that the extranumerary chromosome is a remnant of asynchronous replication, a vestige in a neuroprogenitor that failed to withdraw or complete its replication timing^75^. It has been noted that olfactory neurons singularly and stochastically select one olfactory receptor gene from an array of over a thousand possibilities. The olfactory neuron must then select among which allele of this selected gene for final expression^76^. The allele selection appears to hinge on asynchronous replication; the expressed allele is faithfully marked by early replication. Others have also noted asynchronous replication as a possible epigenetic mark for monoallelic expression^77–79^. In this spirit and along the emerging field of genome imaging, we believe our spatial genome aligner provides a new perspective of chromatin imaging analysis with which we hope to unravel foundational biological principles.

## Methods

### Spatial genome alignment algorithm

Conceptually, we abstract each detected fluorescence signal from a 3D image stack as a four-dimensional (4D) node *v* *=* (*x, y, z, t*). Here, (*x, y, z*) correspond to subpixel spatial coordinates of a genomic locus detected in imaging, with a resultant positional uncertainty (*σ*_*x*_*, σ*_*y*_*, σ*_*z*_) in each spatial axis discovered from 3D Gaussian fitting. The fourth dimension, *t*, corresponds to an order of the gene on the reference genome, ordered from 5′ to 3′ for every chromosome. For notation, we use *v*_*t*;*i*_ to refer to a node *i* with spatial position (*x*_*i*_*, y*_*i*_*, z*_*i*_*)* corresponding to order *t* on the reference genome; there may be as many as *n*_*t*_ detected nodes for a given gene order *t*.

1. Graph construction: we define a directed acyclic graph *G* = (*V, E*) as follows:
  *V* *=* *{v*_*t*;*i*_*}*, *1* ≤ *i* ≤ *n*_*t*_, *1* ≤ *t* ≤ *T* represents the set of nodes in the graph, for every candidate node *i* of a gene order *t* among *n*_*t*_ candidates, for all *T* genes on the reference genome.
    We note that due to signal dropout, there may be genes for which *n*_*t*_ *=* 0, in which case no nodes for the given order *t* are populated.
  *E* *=* {$w_{t;\,i}^{t + c;j}$ *=* ($v_{t;i},v_{t + c;j}$)}, *1* ≤ *i* ≤ *n*_*t*_, *1* ≤ *j* ≤ *n*_*t+c*_*, 1* ≤ *t* ≤ *T, 1* ≤ *c* ≤ *C* represents the set of all edges connecting ordered pairs of nodes, between every candidate node *i* of a gene order *t* among *n*_*t*_ candidates, to every candidate node *j* of a gene order *t* *+* *c* among *n*_*t+c*_ candidates, for allowable skips 1 ≤ *c* ≤ *C*, for all *T* genes on the reference genome.
  We disallow self-loops by enforcing a lower bound on the skip parameter *c* ≥ 1, such that no edges propagate 3′ → 5′, or more explicitly, no edges from a node of order *t* connect to any node with order less than or equal to *t* + 1. This prevents discovery of certain structural variants, such as inversions, translocations and duplications, but helps restrict solutions to strictly the reference genome. We permit nodes to ‘look ahead’ to downstream genes by skipping up to a permissible upper bound *c* ≤ *C*, scaled later by an affine gap penalty. This accounts for signal dropout resulting in false negative signals, in which case all nodes of a given order *t* may be false positives and must be skipped.
2. Calculate bond probabilities: we weight the edges using a physical analogy of a polymer model of DNA. Namely, we use the freely jointed Gaussian chain model, wherein chemical bonds model connections between two monomers. Here, our discrete spatially resolved genomic locations are analogous to these monomers, connected on the same chromatin fiber. In this model, the spatial distance separating these two locations is modeled after a Gaussian distribution:
  4 $$w_{t;\,i}^{t + c;j} = 4\pi (R_{t;i}^{t + c;j})^2\frac{1}{{\left( {2\pi (S_{t;i}^{t + c;j})^2} \right)^{3/2}}}{\mathrm{e}}^{ - \left( {\frac{{(R_{t;i}^{t + c;j})^2}}{{2(S_{t;i}^{t + c;j})^2}}} \right)}$$
  where $R_{t;i}^{t + c;\,j}$ is the distance in nanometers between the *i*th node with gene order *t* to the *j*th node with gene order *t* *+* *c*. $S_{t;i}^{t + c;j}$ is expanded as:
  5 $$(S_{t;i}^{t + c;j})^2 = \sigma _{t;i}^2 + \sigma _{t + c;j}^2 + \frac{2}{3}l_p\tau L_{t;i}^{t + c;j}$$
  where the positional uncertainties of both the start locus $\sigma _{t;i}^2$ and end locus $\sigma _{t + c;j}^2$ are appended to the second moment $S^2 = \frac{2}{3}l_p\tau L_{t;i}^{t + c;j}$. We note these positional uncertainties are previously measured via a Gaussian fitting routine around each detected fluorophore, and done separately for each laser channel. While the positional uncertainties may differ across imaging platforms, we estimate based on Su et al.^30^ that the uncertainty is roughly 50 nm when the drift and chromatic aberration are appropriately corrected for. Operationally, we permit the toggling of the term arising from integrating the spherical differential volume $4\pi (R_{t;i}^{t + c;j})^2.$ In our analysis, we did not use this term, instead opting for the following:
  6 $$w_{t;\,i}^{t + c;j} = \frac{1}{{\left( {2\pi (S_{t;i}^{t + c;j})^2} \right)^{3/2}}}{\mathrm{e}}^{ - \left( {\frac{{(R_{t;i}^{t + c;j})^2}}{{2(S_{t;i}^{t + c;j})^2}}} \right)}$$
  Here, *l*_p_ is the persistence length of DNA in nanometers, *τ* is a scaling factor that converts genomic distance in base pairs to spatial distance in nanometers and $L_{t;i}^{t + c;j}$ is the genomic distance in base pairs that separate the start locus *v*_*t*;*i*_ and end locus $v_{t + c;j}$. The positional uncertainty is calculated separately for the start and end locus to accommodate chromatic aberrations, in which case loci imaged using different laser channels may have different localization error. $\tau L_{t;i}^{t + c;j}$ together reflects the contour length of the DNA polymer. Collectively, this allows a comparison of the observed spatial distance $R_{t;i}^{t + c;j}$ that separate two loci with an estimated spatial distance parametrized as $S_{t;i}^{t + c;j}$.
  In this present study, we estimate *τ* by fitting a power-law function through pairwise spatial distance and genomic interval data to estimate a length scale of each basepair per nanometer, as had been done in previous literature^26,27,31,42^. We do so separately for each chromosome, as previous studies show the length scales differ across mouse chromosomes^31^. We fix the persistence length at *l*_p_ = 150 bp, and allow *τ* to scale both the contour length *L* and the persistence length *l*_p_, reducing one free parameter. Our key assumption in this model relies on the conversion factor *τ*. DNA compaction is known to change throughout A/B compartments^7^ and highly expressed genes^80^ among other regions, and our model’s genomic scale is fixed: it does not recapitulate the exact spatial distance at each imaged locus. Its fidelity may also change at different genomic scales (that is, 1 Mb, 25 kb, 5 kb). However, our main aspiration is to use this estimated spatial distance to inform soft decisions in the selection of spots, in addition to optical qualities of signals detected, in a bid to trace chromatin structures.
  In this manner, a traversal from chromosome start to end along this graph would accumulate a sequence of bond probabilities whose product reflects the physical likelihood of the discovered polymer:
  7 $${\mathrm{CDF}} = \mathop {\prod }\limits_{h = 1}^H w_{v_h}^{v_{h + 1}},p = \left\langle {v_1,\,v_2 \ldots v_H} \right\rangle$$
  for every node *v* visited on path *p* from source to sink.
  Each edge weight $w_{t;\,i}^{t + 1;j}$ is negative log normalized into positive edge weights whose additive sum is equivalent to the polymer CDF. We do so for several reasons: (1) this controls for numerical underflow in calculating the multiplicative product of small decimals; (2) this reframes the optimization objective from maximizing the likelihood function to minimizing the negative log likelihood and (3) the CDF is now computed as a sum of edge weights, permitting the use of existing dynamic programming shortest-path algorithms solving for additive edge weights. Below, we write edge weights *w* to represent negative log normalized bond probabilities. To permit nodes to skip potential false positive and ‘look ahead’ to downstream genes, we apply penalize each bond skipped:
  8 $$w\prime _{t;\,i}^{t + c;j} = \gamma _{c - 1}\,w_{t;\,i}^{t + c;j}$$
  where *γ*_*c*-1_ is a gap penalty scaled for every skip *c*. Adjacent nodes (*c* = 1) are not penalized. This gap penalty is an important regularization policy that makes sure a skipped edge never has a smaller cost than the cumulative cost of an equivalent number of consecutive edges. For example, if a segment of 1 Mb has a log-bond probability of 5,013.34 (0.34 nm per bp, *l*_p_ = 150 bp, 1 pixel is roughly 100 nm) and a segment of 2 Mb has a log-bond probability of 10,014.38, the cost of ‘skipping’ an edge (traversing a 2 Mb bond) may be preferred instead of traversing two consecutive 1 Mb edges (2 × 5,013.34 = 10,026.68). We apply a multiplicative gap penalty of at least this ratio $\gamma _1 \ge \frac{{10026.68}}{{10014.38}}$ to prevent a preferential skip.
3. Initialize adjacency matrix: we append a single source and a single sink node in our graph to allow gaps in the start and end of polymer alignment. From our graph *G* with total $N = \mathop {\sum }\limits_{t = 1}^T n_t$ nodes, we construct an (*N*, *N*) adjacency matrix that is padded by an additional row with index 1 and column with index *N* + 2 to an (*N* + 2, *N* + 2) matrix. We initialize the first row of the adjacency matrix with ‘pseudo’-bonds that enable up to the first *K* genes to be skipped. These edges linking an imaginary starting position to an observed position are weighted as:
  9 $$w_{1;\,i}^{k;j} = \frac{1}{{\left( {\frac{{4\pi }}{3}l_p\tau L_{t;i}^{t + c;j}} \right)^{3/2}}}{\mathrm{e}}^{ - \left( {\frac{{\alpha ^2\tau L}}{{\frac{4}{3}l_p}}} \right)},\,1 < k \le K$$
  where *α* scales an imaginary stretched genomic segment with an implicit skip penalty.
  We also initialize the last column of the adjacency matrix with ‘pseudo’-bonds that enable up to the last *K* genes to be skipped. Similarly, the edges linking an observed position to an imaginary ending position is weighted as:
  10 $$w_{N - k;\,i}^{N;j} = \frac{1}{{\left( {\frac{{4\pi }}{3}l_p\tau L_{t;i}^{t + c;j}} \right)^{3/2}}}{\mathrm{e}}^{ - (\frac{{\alpha ^2\tau L}}{{\frac{4}{3}l_p}})},\,1 < k \le K$$
  These initializations behave as ‘seeds’ that permit late chromosome starts (from locus 1 − *k*) and early chromosome ends (from locus *N* − *k* − *N*) should the first or last few loci have poor labeling efficiency and need to be skipped.
4. Path finding: we find the shortest path from source to sink in our graph using a dynamic programming algorithm. As all edge weights in this directed graph are nonnegative, and the sum of traversed edges equate to the CDF of the traversed polymer, we use Dijkstra’s shortest-path algorithm as a dynamic programming means for finding the most plausible polymer:

11 $$\min \mathop {\sum }\limits_{h = 1}^H w_{v_h}^{v_{h + 1}},\,p^ \ast = \left\langle {v_1,\,v_2 \ldots v_H} \right\rangle$$

for every node *v* visited on the shortest path p^*^.

We estimate our worst-case time complexity to be:

12 $$\Theta \left( {\left| E \right| + \left| V \right|\log \left| V \right|} \right) = \Theta \left( {\left| {\mathop {\sum }\limits_{t = 1}^T n_t\mathop {\sum }\limits_{t + 1}^{t + C \le T} n_t} \right| + \left| {\mathop {\sum }\limits_{t = 1}^T n_t} \right|\log \left| {\mathop {\sum }\limits_{t = 1}^T n_t} \right|} \right)$$

### Polymer fiber karyotyping algorithm

**Extended Data Fig. 10:**
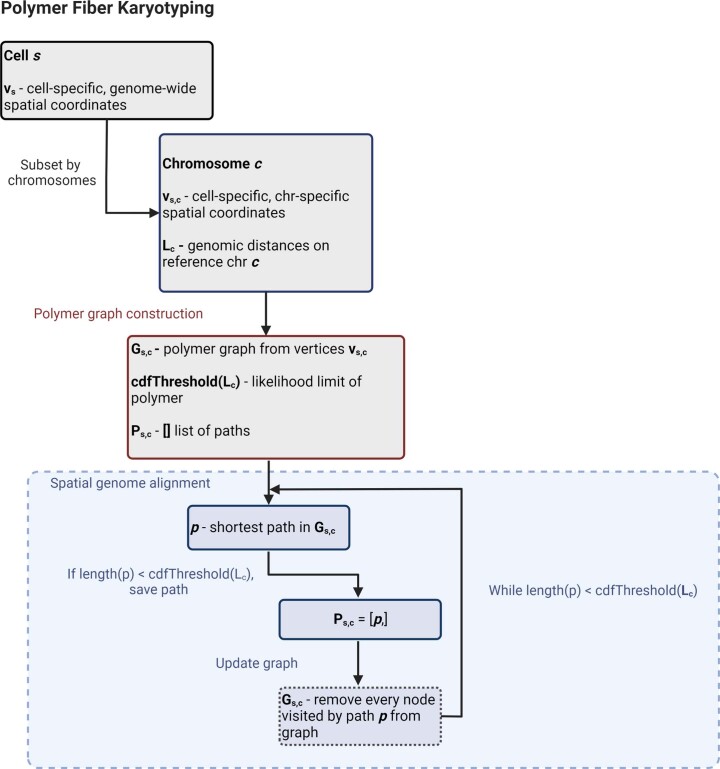
Polymer fiber karyotyping routine. Workflow of iterative spatial genome alignment to uncover all physically likely fibers of a given chromosome in single cells. For any given cell, after subsetting detected loci by chromosomes, the aligner constructs a directed acyclic graph (DAG) from all signals belonging to a given chromosome. The aligner next finds the shortest path through this DAG using dynamic programming, where this path represents a putative polymer fiber and the path score its physical likelihood. For every putative fiber discovered from alignment, the aligner updates the DAG by subtracting all visited nodes while the discovered fiber has a likelihood score within threshold. This process is repeated until no polymer under threshold can be discovered, upon which the routine terminates alignment.

We developed a routine that finds all possible polymers of a given chromosome on a cell-by-cell basis. Chiefly, we accept all polymers below a physical likelihood threshold. This threshold can be derived by scrambling the genomic intervals separating each probed locus, such that the observed genomic distance no longer abides by the expected distances. We then perform an iterative search wherein nodes of each shortest path discovered are subtracted from graph *G* before searching for the next shortest path, until no likely paths below the physical likelihood threshold can be discovered (Extended Data Fig. 10).

We retrieved spatially resolved fluorescent signal information for mouse genome-wide DNA seqFISH+ probe sets at multiple length scales (1 Mb, 25 kb), and for multiple cell types (mESC https://zenodo.org/record/3735329; mouse cortical neurons 10.5281/zenodo.4708112). Congruent with previous publications^26–32,42^, we fit a power-law function through pairwise spatial distances of observed loci, plotted against its genomic-distance separation. From this power-law function, we estimated a parameter corresponding to nanometers per basepair, for every cell type, for every mouse chromosome. To evaluate the performance of our parameter in its ability to recapitulate true chromatin structure, we performed a hyperparameter search. Explicitly, we fit a power function through pairwise spatial and genomic intervals, and with the power function we estimated the distance scale (nanometers per basepair) for a range of different genomic resolutions (that is, 10 kb, 100 kb, 1 Mb, 10 Mb …) (Extended Data Fig. 3a). Using 10% of cells in each dataset, we performed spatial genome alignment and compared its median distance matrix to Hi-C or cell-type resolved Dip-C. We used the parameter that engendered the best fit for our final analysis.

With this spatial distance parameter, for every nucleus and for every chromosome, we iteratively performed spatial genome alignment until no physically plausible fibers could be discovered. Finally, we counted the number of fibers discovered for every chromosome to assign a chromosome copy number, producing a cell karyotype.

### mESC Hi-C data analysis

To evaluate the spatial genome alignment results of mESCs, we retrieved mESC Hi-C contact matrices from the 4DN Data Portal (experiment set 4DNESU4Y9CBF). Next, we used Straw (https://github.com/aidenlab/straw, v.0.0.6) to extract Knight–Ruiz normalized count matrices for every mouse. Whereas read counts can be evenly binned, the loci imaged by DNA seqFISH+ spanned irregular intervals. To compare Hi-C with DNA seqFISH+ imaging data, where the genomic distances separating each locus are irregular intervals, we performed the following normalization. For DNA seqFISH+ imaging data, we kept the imaged locus ordered 5′ to 3′ closest to each integer 1 Mb or 25 kb bin, dropping all other loci to calculate a final distance matrix. For the corresponding Hi-C matrix, we binned the reads at 1 Mb and 25 kb resolution, respectively, dropping the same bins removed from the DNA seqFISH+ distance matrix. To assess spatial genome alignment accuracy, we compared the median imaging distance matrix to its corresponding KR-normalized Hi-C contact matrix using the Spearman correlation coefficient.

### Excitatory mouse cortical neuron Dip-C data analysis

To evaluate the spatial genome alignment results of excitatory mouse cortical neurons, we retrieved cell-type resolved Dip-C contact matrices from NCBI GEO (accession no. GSE162511)^74^. First, we note a dearth of multi-modal data that ideally allows concomitant cell-type classification using one sequencing modality and proximity-ligation analysis on the same cell. In Dip-C, neuronal cell types were resolved by coprojecting *NeuN*+ neurons with bulk Hi-C sequencing of multiple cell types. Neurons coclustering with *Slc17a7+* cells delineating excitatory neurons were also classified as excitatory. It is possible some of these cells classified as excitatory may belong to broader cell types. In contrast, DNA seqFISH+ imaging resolved both RNA and DNA in the nuclei, which allowed excitatory neuron markers (that is, *Slc17a7+*, *Neurod2*) detected in RNA imaging to label cell types. Second, we note a difference in the mouse strains of the two datasets. Dip-C focused on an F1 cross with CAST/EiJ × C57BL/6J background while the DNA seqFISH+ imaging purely focused on C57BL/6J mice.

Amid these caveats, we used Straw to extract Knight–Ruiz normalized count matrices for every mouse chromosome. For the DNA seqFISH+ imaging data, we again kept the imaged locus ordered 5′ to 3′ closest to each integer 1 Mb bin, dropping all other loci to calculate a final distance matrix. For the corresponding Dip-C contact matrix, we binned the reads at 1 Mb resolution and dropped the same bins removed from the imaging distance matrix. To assess spatial genome alignment accuracy, we compared the imaging distance matrix to its corresponding Hi-C matrix using the Spearman correlation coefficient.

Using the same Dip-C dataset, we inspected haplotype-resolved reads—namely preprocessed ‘seg’ files—to evaluate read counts assigned to each haplotype. We discarded any ambiguous or multi-contact read pairs and counted cells wherein one haplotype has nearly twice as many reads as the other, as a proxy for identifying copy number variations and the haplotype source of those copy number variations at the single neuron level.

### Benchmarking against M-DNA-FISH spot selection algorithm

In conjunction, we analyzed M-DNA-FISH imaging of a 210-kb genomic region spanning the Sox2 locus (chr3:34,601,078-34,811,078) based on previous work^49^. We considered all chromosome centers assigned to the 129 allele, which lacks the 7.5 kb tandem CTCF-binding sites inserted on the CAST allele. The previous E–M routine outlined in refs. ^28,49^ generates ten candidate spots per locus, per putative chromosome in every nucleus, assuming a diploid cell line. Each candidate spot is assigned a score, derived as a combination of: (1) fluorescence intensity, (2) proximity to chromosome center and (3) agreement with moving average of previous loci positions. A candidate spot with a score of −1.5 or more is considered a high-quality spot for the E–M routine, with more negative scores tracking with decreasing quality. For spatial genome alignment, we fed all candidate spots with a much lower quality threshold score of −4, such that for every high-quality spot there is also a low-quality spot. We included this extra noise and ignored the fluorescence intensity information to demonstrate the supreme use and specificity of genomic distances as a spot selection criterion. We compared the spatial genome alignment results and E–M tracing results against Hi-C, which sequenced the control mouse chr3 lacking the CTCF-binding site insertion. We also calculated the Spearman correlation between the discovered pairwise median distances and pairwise KR-normalized Hi-C contact frequency for each of the two algorithms.

### Homolog assignment and sister chromatid aggregation analysis

In diploid cells, we used density-dependent clustering algorithm DBSCAN^81^ (scikit-learn v.1.0.1) to separate homologous chromosomes residing in separate chromosome territories.

In tetraploid cells, instead of using density-based clustering to parse and assign homologs, we opted to take a different approach. We first assigned chromatin interaction patterns (that is, separate homologs, compact homologs, separated sisters in tetraploid cells) with DBSCAN to find spatially separable structures and classify tetraploid cells. To assign sister chromatids and in turn homologs, we paired chromatin fibers by the closest starting positions and assigned them as sisters of the same homolog. This allowed us to pair sisters as part of the same homolog in tetraploid cells that had only one spatially dense cluster (that is, compact homologs). In the setting of compact chromosomes where two homologs are spatially not separable, we accounted for alternative pairing scenarios, such as pairing by the telomeric ends. We analyzed the spatial separation of each chromosome starts and ends based on pairing by centromeric starts as well as pairing by telomeric ends, and all possible alternative pairings.

### Sister chromatid misselection assessment

To identify potential loci belonging to one sister chromatid but misselected by the other, we examined all 402 putative sister chromatid pairs for mESC chromosome 1 (177 loci in length). First, we interpolated spatial coordinates for any loci not detected on a sister chromatid fiber. Next, using a sliding window of three loci beginning at locus 3 and sliding until locus 175, we exchanged spatial positions between the main sister and attendant sister for these three loci. Finally, we recalculated the bond probabilities of three loci upstream to the three loci exchanged. If the multiplicative product of all recalculated probabilities is more likely than the product of original bond probabilities, we call this a misselection event. We also counted any consecutive sequence of misselections and called this a cross-over event.

### Simulation of aneuploid cells and analysis

To study the impact our aligner has on chromosome counting in isolation from other sources of technical artifacts, we used the polychrom package (https://github.com/open2c/polychrom/) to simulate chromosomes under nuclear confinement^82^. We randomized copy numbers for each of the 20 mouse chromosomes, generating 1,000 such sets of aneuploid cells. We then performed polymer fiber karyotyping on each cell, repeating the process for different levels of added noise and signal dropout. Detailed descriptions of the parameters underlying the simulation, as well as descriptions of the analysis can be found under Supplementary Materials.

### Simulation of sister chromatid pairs and analysis

To study the impact our aligner has on parsing tightly paired chromatin fibers from other sources of technical artifacts, we used the polychrom package to simulate tightly paired chromatin fibers. Simulating 100 pairs of sister chromatids corresponding to mouse chromosome 1, we varied the pairing strength between coupled fibers to generate five separate conditions. We then applied polymer fiber karyotyping on each of the 100 pairs, across the five conditions, fixing the signal dropout to 50% and added noise to 0%. Detailed descriptions of the parameters underlying the simulation, as well as descriptions of the analysis can be found under Supplementary Materials.

### Normalization of chromatin levels for pseudotime analysis

Consistent with previously published work, we constructed a generalized linear model for normalizing sequential immunofluorescence data. In this generalized linear model are latent variables controlling for and removing contributions from cell size and total fluorescence intensity over all chromatin marks, as well as batch effect from replicate ID and field of view. Specifically, we used a noncanonical log-link function:

13 $$\log Y_i \approx \beta _0 + \mathop {\sum }\limits_j \beta _jX_j$$

where *Y*_*i*_ is the vector of total fluorescence intensity of chromatin mark *i* across all cells, and *X*_*j*_ are latent variables contributing to bias. Using the Pearson residuals of each fitting, we corrected the readout of two chromatin marks, H4K20me1 and H4K16ac, with which we constructed a principal curve to order cells across the cell cycle or pseudotime. Matching previously published results, H4K20me1 and H4K16ac peaked at opposite ends of the pseudotime axis, ordering cells from G2/M.

### Gene dosage and transcriptional activity analysis

After polymer fiber karyotyping, we stratified cells by the copy numbers discovered for each chromosome and inspected the seqFISH+ RNA imaging corresponding to genes in the DNA seqFISH+ imaging. We performed pairwise *t*-tests to evaluate statistical significance for the few genes that lie on each mouse chromosome.

### Reporting summary

Further information on research design is available in the Nature Portfolio Reporting Summary linked to this article.

## Online content

Any methods, additional references, Nature Portfolio reporting summaries, source data, extended data, supplementary information, acknowledgements, peer review information; details of author contributions and competing interests; and statements of data and code availability are available at 10.1038/s41587-022-01568-9.

## Supplementary information

Supplementary Information Extended details of simulation parameters using polychrome to generate synthetic aneuploid cells and paired chromatin fibers.

Reporting Summary

## Data Availability

All traced chromatin structures with spatial genome alignment are available and hosted on 4D Nucleome Data Portal (accession nos. 4DNFIXGTJBGU, 4DNFIFBXKXK9, 4DNFIYNWVJEP, 4DNFI7G3BWDF, 4DNFIVBL8AWT, 4DNFIU73OR5W and 4DNFIS6MLXGA). Published seqFISH+ datasets analyzed in this paper are hosted and available on Zenodo (mESC https://zenodo.org/record/3735329; mouse cortical neurons 10.5281/zenodo.4708112). Published M-DNA-FISH datasets analyzed in this paper are hosted and available on 4D Nucleome Web Portal (accession nos. 4DNESIGBIXQS, 4DNESC5PKTQ9, 4DNES51KSIZ9, 4DNESNEKOCAP and 4DNESOXQX1JT).

## Code availability

The spatial genome aligner is available at https://github.com/b2jia/jie (ref. ^83^). The aligner and peripheral analysis rely on algorithms implemented in scipy (v.1.7.2)^84^ and scikit-learn (v.1.0.1)^81^. A walkthrough of the intuition behind this algorithm can be found at https://github.com/b2jia/jie/blob/main/jupyter/00-spatial-genome-alignment-walk-thru.ipynb.
