# Author Correction: A spatial genome aligner for resolving chromatin architectures from multiplexed DNA FISH

**DOI:** 10.1038/s41587-023-01673-3

**Published:** 2023-01-16

**Authors:** Bojing Blair Jia, Adam Jussila, Colin Kern, Quan Zhu, Bing Ren

**Affiliations:** 1 Bioinformatics and Systems Biology Graduate Program, University of California San Diego, La Jolla, CA USA; 2 Medical Scientist Training Program, University of California San Diego, La Jolla, CA USA; 3 Department of Cellular and Molecular Medicine, Center for Epigenomics, University of California San Diego, La Jolla, CA USA; 4 Ludwig Institute for Cancer Research, La Jolla, CA USA; 5 Institute of Genomic Medicine, Moores Cancer Center, School of Medicine, University of California San Diego, La Jolla, CA USA

**Keywords:** Gene regulation, Image processing

Correction to: *Nature Biotechnology* 10.1038/s41587-022-01568-9. Published online 2 January 2023.

In the version of this article initially published, the 4D Nucleome Web Portal accession number (4DNESU4Y9CBF) provided in the Data availability section was incorrect and has been replaced with accession numbers 4DNESIGBIXQS, 4DNESC5PKTQ9, 4DNES51KSIZ9, 4DNESNEKOCAP and 4DNESOXQX1JT in the HTML and PDF versions of the article.

