# Supplementary material for: A spatial genome aligner for resolving chromatin architectures from multiplexed DNA FISH
Source: Nat Biotechnol. 2023 Jan 2;41(7):1004–17. doi: 10.1038/s41587-022-01568-9 (PMC10344783; doi:10.1038/s41587-022-01568-9)
Supplement: Supplementary file 1 — Extended details of simulation parameters using polychrome to generate synthetic aneuploid cells and paired chromatin fibers. [file 41587_2022_1568_MOESM1_ESM.pdf]

nature biotechnology

Article

https://doi.org/10.1038/s41587-022-01568-9

A spatial genome aligner for resolving chromatin architectures from multiplexed DNA FISH

In the format provided by the authors and unedited

Simulation of aneuploid cells and subsequent analysis

To study the impact our aligner has on chromosome counting in isolation from other sources of technical artefact, we utilized the polychrom package (https://github.com/open2c/polychrom/) to simulate chromosomes under nuclear confinement[81].

Using Polychrom's `forcekits.polymer_chains` module, we generated randomized copy numbers – ranging from 0, 1, 2, 3, or 4 copies – for each of the 20 chromosomes that span the mouse genome. This module generates multiple chains, and permits forces to be communicated between different chains without explicitly instantiating each chromatin fiber in isolation. For each chain, we downsampled the genomic resolution of each monomer step to 100 kb so that the total cumulative monomers fit within the allowable limit (~10^5 monomers). In so doing, some of the genomic positions imaged in seqFISH+ experiments lost specificity and became "merged" in the same 100 kb "bin". For instance, whereas 177 distinct loci were imaged for chr1, downsampling our polymers to 100 kb resolution resulted in 171 distinct loci. We discarded any spots not belonging to an imaged bin as input for spatial genome alignment, utilizing the same reference genome as was designed for seqFISH+ 1Mb resolution tracing. We permitted a relatively stiff chromatin fiber (`angle_force` → k:6) in our simulations, corresponding to a persistence length of 6 bonds (600 kb). From experimental data, we calculated the average cosine angle between polymer bonds and fitted an exponential function against genomic distance to estimate a persistence length. This estimated persistence length of imaged chromosomes are on the order of several Mb (seqFISH+ mouse chr1 : 7.897 Mb), which may be scale dependent and subject to experimental and fitting error. We note that 7.897 Mb exceeds Polychrom's permitted persistence length up to 10 times a bond length, which is set at 100 kb.

To enforce chromosome territories, we used Poisson sphere sampling to generate roughly equidistant chromosome territory “centers” scattered within a sphere standing in place for a nucleus. About each center, we prescribed spherical confinement to put one chain inside the given territory. Finally, we enforce an overall spherical confinement to emulate the nuclear envelope, confining all spherically-confined territories together. We realize chromosome territories may not be spherical in nature, and note this as one caveat of our simulated system.

While we enforced spherical chromosome territories, we permitted some weak trans-fiber interactions (`nonbonded_force` → trunc:8) by limiting the nonbonded repulsive forces. This allowed some fibers to pass through another territory and intermingle; we show some representative images of such fibers in Supplemental Fig. 3A, leftmost column. In such scenario, should our aligner select a true signal that instead belongs to a different intermingling fiber, we also considered this a “False Positive” event. Explicitly, “False Positive” encompasses two scenarios: the selection of stray localizations added as noise, and the selection of true signals intended to be selected by a different fiber.

We also note that the intra- and inter-chromosomal distances between loci does not recapitulate chromatin structures observed in neither Hi-C nor seqFISH+ imaging. We did not use any contact matrices as input for the simulation, and is one limitation of our simulation. We considered intra- and inter-chromosomal distances in units of bonds, assuming each bond represents a 100kb step. We rescaled our distance parameters accordingly.

At scale, we simulated 1000 such cells of randomized karyotype, as shown in Supplemental Fig. 3A, headline "Karyotype". We randomized copy number for each chromosome so that for a given cell, there is little correlation between copy number of one chromosome and that of another chromosome. For each simulated cell, we created a grid of 49 different conditions, spanning combinations of 7 different false negative rates (FNR; 0%, 20%, 40%, 50%, 60%, 80%, 100%) and 7 different false positive rates (FPR; 0%, 20%, 40%, 50%, 60%, 80%, 100%).

We defined false negative rate as the proportion of dropouts *per chromatin fiber*. For example, on mouse chr1 for which there were 177 imaged loci on each chromatin fiber, a 40% FNR would correspond to 106 loci kept while 71 loci are randomly selected from each fiber for omission. We did not synchronize which loci are dropped for a given chromosome, as might be encountered in a real experiment (ie. poor probe design for a locus might lead to dropout for every chromatin fiber). This means that the location of missing loci is different for multiple copies of a given chromosome.

We defined false positive rate as the proportion of all loci with *one* extra signal. This is a departure from previously defined false positive rates. In Takei *et al.*[31] and Takei *et al.*[32], false positives were defined as detection of unused barcodes in a codebook that spans genome wide. They detected a median 0.87 ± 0.76% false positive rate (32 ± 27.8 spots; 3660 labeled loci). By our estimate, this is around 1 spot belonging to 1-2 loci per chromosome assuming uniform error coverage. Therefore, we simulated 1 extra signal for a proportion of all loci on a given chromosome. For example, on mouse chr1 for which there were 177 imaged loci on each chromatin fiber, a 20% FPR would correspond to 35 loci bearing an extra signal. We simulated

the extra signals by performing Poisson sphere sampling to generate noisy signals that are spatially uniform throughout the entire nucleus. We note that our lowest non-zero FPR (20%) would correspond to 472 spots (2398 total loci), at least one order of magnitude more than what has been experimentally observed in seqFISH+ imaging.

Using imperfect information – namely pairwise genomic and spatial distances observed with 50% FNR and 20% FPR – we randomly selected 10 cells through which we fit a power-function to estimate a distance parameter. In a real experiment, we rationalize our choice of a distance parameter as one that achieves the highest Spearman correlation against bulk Hi-C data. In our simulation, we lack a conserved contact frequency matrix to compare with. We instead relied on feedback of labels to calculate true-coverage rate, false-positive rate and false-negative rate to find the distance scale that minimizes these sources of error. We note that we only fit on the first 10 cells, under imperfect data, and utilized this distance function for all 49 conditions of noise.

After fitting a distance function, we performed our polymer fiber karyotyping routine on all 1000 cells, each repeated 49 times under different noise conditions. We parsed the signals selected by our spatial genome aligner using the following protocol (Supplemental Fig. 3A, right):

a. We considered any true positive signal selected, in the presence or absence of (a) true positive signals belonging to other polymer fibers of the same locus on the same chromosome or (b) false positive signals of the same locus on the same chromosome injected into the simulation, as <u>True Coverage (True Positive → True Positive)</u>.
b. We considered any false positive signal selected – be it (a) true positive signals belonging to other polymer fibers of the same chromosome or (b) false positive signals injected into the

simulation – in the *absence of true signal* at the given locus on the present fiber considered, as Type I Error (True Negative → False Positive).

- This allows us to evaluate if our spatial genome aligner has an *anchoring bias* (selecting a false positive spot rather than skipping a spot)

c. We considered any false positive signal selected – be it (a) true positive signals belonging to other polymer fibers of the same chromosome or b) false positive signals injected into the simulation – in the *presence of true signal* at the given locus on the present fiber considered, as Type II Error (True Positive → False Positive).

- This allows us to evaluate if our spatial genome aligner's resiliency in parsing noisy signals from true detected signals.

d. We considered any true positive signals skipped in the presence of true signal at the given locus on the present fiber considered, as Type III Error (True Positive → False Negative).

- This allows us to evaluate if our spatial genome aligner's gap penalty favors skipping or not.

e. We considered any true negative signals skipped in the absence of true signal at the given locus on the present fiber considered, as True Dropout (True Negative → True Negative).

- This allows us to evaluate if our spatial genome aligner's gap penalty is correctly weighted to enable skipping.

We summarized these categorized loci selection at three resolutions (a) at the locus level for a given chromosome under a specified noise condition (b) across all chromosomes under a specified noise condition (c) across all noise conditions.

Simulation of sister-chromatid pairs and analysis

We simulated 100 pairs of sister chromatids of mouse chromosome 1, subject to different strengths of pairing and different levels of noise. To inform parameters of our analysis, we investigated mouse chromosome 1 in seqFISH+ whole genome imaging at 1 Mb scale, and measured key properties of chromatin fibers at this resolution. Specifically, we measured (Supplemental Fig. 7A):

- The inter-sister distance for every matched locus (median distance = 1606.80nm)
- The intra-sister distance scale for every adjacent loci (1.047 x 10-5 nm/basepair). This translated to 1047.65 nm for a 1 Mb separation.
- The persistence length of chromosome 1 at 1 Mb resolution (l_p = 7897.47 kb).
- The pairing strength – how faithfully one sister shadows another – in terms of Spearman correlation of spatial positions (μ = 0.7843).

Because polychrom permits a persistence length 10 times the step size, we resorted to downsampling genomic resolution such that each monomer step is equivalent to 1 Mb. In so doing, some of the genomic positions imaged in seqFISH+ experiments lost specificity and became "merged" in the same 1 Mb "bin". For instance, whereas 177 distinct loci were imaged for chr1, downsampling our polymers to 1 Mb resolution resulted in 133 distinct loci. We discarded any spots not belonging to an imaged bin as input for spatial genome alignment, utilizing the same reference genome as was designed for seqFISH+ 1Mb resolution tracing. We utilized a stiffness of 8 Mb (`angle_force`: k=8) to achieve a similar stiffness. Finally, to pair sister chromatids together, we added constant force bonds between matching loci on each pair of sister chromatids. We stipulated the sister pairing bond length to be 1.534 times each polymer bond length (1606.8 / 1047.65).

To study how pairing strength affects our spatial genome aligner's ability to parse two fibers apart, we added sister pairing bond lengths at different frequencies (Supplemental Fig. 7B). Adding every bond, 3, 5, 7 and 9 bonds allowed us to generate a spectrum pairing strengths, with average Spearman correlations ranging from 76.90%, 74.15%, 70.26%, 53.60%, and 42.68% respectively. To emulate experimental conditions we randomly dropped out 50% of loci (50% FNR) on each sister chromatid (Supplemental Fig. 7C, gray).
