# Supplementary material for: A spatial genome aligner for resolving chromatin architectures from multiplexed DNA FISH
Source: Nat Biotechnol. 2023 Jan 2;41(7):1004–17. doi: 10.1038/s41587-022-01568-9 (PMC10344783; doi:10.1038/s41587-022-01568-9)
Supplement: Supplementary file 2 — Reporting Summary [file 41587_2022_1568_MOESM2_ESM.pdf]

nature portfolio

Corresponding author(s): Bing Ren

Last updated by author(s): Oct 9, 2022

Reporting Summary

Nature Portfolio wishes to improve the reproducibility of the work that we publish. This form provides structure for consistency and transparency in reporting. For further information on Nature Portfolio policies, see our Editorial Policies and the Editorial Policy Checklist.

Statistics

For all statistical analyses, confirm that the following items are present in the figure legend, table legend, main text, or Methods section.

n/a	Confirmed	
☒	☐	The exact sample size (*n*) for each experimental group/condition, given as a discrete number and unit of measurement
☒	☐	A statement on whether measurements were taken from distinct samples or whether the same sample was measured repeatedly
☒	☐	The statistical test(s) used AND whether they are one- or two-sided<br>*Only common tests should be described solely by name; describe more complex techniques in the Methods section.*
☒	☐	A description of all covariates tested
☒	☐	A description of any assumptions or corrections, such as tests of normality and adjustment for multiple comparisons
☒	☐	A full description of the statistical parameters including central tendency (e.g. means) or other basic estimates (e.g. regression coefficient) AND variation (e.g. standard deviation) or associated estimates of uncertainty (e.g. confidence intervals)
☒	☐	For null hypothesis testing, the test statistic (e.g. *F*, *t*, *r*) with confidence intervals, effect sizes, degrees of freedom and *P* value noted<br>*Give P values as exact values whenever suitable.*
☒	☐	For Bayesian analysis, information on the choice of priors and Markov chain Monte Carlo settings
☒	☐	For hierarchical and complex designs, identification of the appropriate level for tests and full reporting of outcomes
☒	☐	Estimates of effect sizes (e.g. Cohen's *d*, Pearson's *r*), indicating how they were calculated

Our web collection on statistics for biologists contains articles on many of the points above.

Software and code

Policy information about availability of computer code

Data collection	N/A
Data analysis	We developed a spatial genome aligner which we call `jie`, written in in Python (version 3.8), available at https://github.com/b2jia/jie.<br><br>The spatial genome aligner and peripheral analysis relies on algorithms (ie. DBSCAN) implemented in scikit-learn (version 1.0.1), as well as scipy (version 1.7.2).<br><br>To analyze Hi-C data, we downloaded Hi-C data from accessions listed above, and utilized Straw (https://github.com/aidenlab/straw; version 0.0.6) to extract Knight-Ruiz normalized count matrices for every mouse chromosome.

For manuscripts utilizing custom algorithms or software that are central to the research but not yet described in published literature, software must be made available to editors and reviewers. We strongly encourage code deposition in a community repository (e.g. GitHub). See the Nature Portfolio guidelines for submitting code & software for further information.

Data

Policy information about availability of data

All manuscripts must include a data availability statement. This statement should provide the following information, where applicable:

- Accession codes, unique identifiers, or web links for publicly available datasets
- A description of any restrictions on data availability
- For clinical datasets or third party data, please ensure that the statement adheres to our policy

All traced chromatin structures with spatial genome alignment is available and hosted on 4D Nucleome Data Portal (accessions: 4DNFIXGTJBGU , 4DNFIFBXKXK9, 4DNFIYNWVJEP, 4DNFI7G3BWDF, 4DNFIVBL8AWT, 4DNFIU73OR5W, 4DNFIS6MLXGA).

Prior to spatial genome alignment, we downloaded seqFISH+ whole genome imaging of mouse ESCs from https://zenodo.org/record/3735329, tracing chromatin at both 1 Mb and 25 Kb resolution. To compare mESC chromatin configuration, we downloaded mESC Hi-C data from 4DN Data Portal (accession 4DNESU4Y9CBF).

We downloaded seqFISH+ whole genome imaging of the mouse cortex from https://doi.org/10.5281/zenodo.4708112, tracing at 1 Mb resolution. To compare mouse neuron chromatin configuration, we downloaded cell-type resolved Dip-C sequencing of mouse cortex from NCBI GEO (accession GSE162511).

To benchmark our spatial genome aligner, we analyzed sequential multiplexed DNA-FISH imaging of mouse Sox2 locus available at NCBI GEO (accession GSE153403). To compare our chromatin configuration, we used Hi-C sequencing of the B allele lacking any tandem CTCF insertion as a reference. This Hi-C data is available under the same accession. This multiplexed DNA-FISH data is also available at 4DN Data Portal (accession 4DNESU4Y9CBF).

Human research participants

Policy information about studies involving human research participants and Sex and Gender in Research.

Reporting on sex and gender	N/A
Population characteristics	N/A
Recruitment	N/A
Ethics oversight	N/A

Note that full information on the approval of the study protocol must also be provided in the manuscript.

Field-specific reporting

Please select the one below that is the best fit for your research. If you are not sure, read the appropriate sections before making your selection.

☒ Life sciences ☐ Behavioural & social sciences ☐ Ecological, evolutionary & environmental sciences

For a reference copy of the document with all sections, see nature.com/documents/nr-reporting-summary-flat.pdf

Life sciences study design

All studies must disclose on these points even when the disclosure is negative.

Sample size	We analyzed all seqFISH+ imaged mESCs (n=1160 cells).We analyzed only fully segmented excitatory mouse cortical neurons (n=458 cells) that were collected from the middle z-sections.<br><br>For multiplexed DNA-FISH imaging of mouse Sox2 locus, we focused on the B allele lacking any tandem CTCF insertions. We filtered for cells (n=366) where at least 35 of 41 rounds of hybridization had detectable signal.
Data exclusions	While there were 2762 neurons in total imaged in seqFISH+ experiments on mouse brain tissue sections, we focused on 701 fully segmented neurons in the middle z-sections, and of these 701 we elected to analyze the most dominant cell type, excitatory neurons (n=458).<br><br>For multiplexed DNA-FISH imaging of the Sox2 locus, we analyzed only the B allele lacking any tandem CTCF insertions and not the A allele bearing the insertion. We also filtered for cells (n=366) where at least 35 of 41 rounds of hybridization had detectable signal.
Replication	We merged all four mESC seqFISH+ experiments (cumulatively n=1160 cells), each spanning multiple fields of views. Separately, we merged all three mouse brain seqFISH+ experiments, each spanning multiple fields of views.<br><br>For our benchmarking analysis utilizing multiplexed DNA-FISH experiments of the Sox2 locus, we utilized one imaging experiment spanning multiple fields of views.

Randomization	As reported previously, seqFISH+ experiments selected individual cells, spanning 5 to 10 different fields of views, randmoly chosen for image acquisition for each experiment.
Blinding	No blinding was required for this study, as was not required for seqFISH+ imaging of mESCs and mouse brain. Our spatial genome aligner is an automated algorithm.

Reporting for specific materials, systems and methods

We require information from authors about some types of materials, experimental systems and methods used in many studies. Here, indicate whether each material, system or method listed is relevant to your study. If you are not sure if a list item applies to your research, read the appropriate section before selecting a response.

Materials & experimental systems

n/a	Involved in the study
☒	☐ Antibodies
☒	☐ Eukaryotic cell lines
☒	☐ Palaeontology and archaeology
☒	☐ Animals and other organisms
☒	☐ Clinical data
☒	☐ Dual use research of concern

Methods

n/a	Involved in the study
☒	☐ ChIP-seq
☒	☐ Flow cytometry
☒	☐ MRI-based neuroimaging
